# Dual antimicrobial and anti-inflammatory efficacy of cetylpyridinium–cromolyn ion-pair nanoparticles delivered *via* modified gelatin

**DOI:** 10.1039/d6ra07879f

**Published:** 2026-11-04

**Authors:** Sukirti Dobriyal, Sarvjeet Das, Juhi Saxena, Anupam Jyoti, Prashant Shukla

**Affiliations:** a Department of Pharmaceutical Sciences, School of Health Sciences and Technology, UPES Bidholi, via Prem Nagar Dehradun-248007 Uttarakhand India prashukla@outlook.com Prashant.shukla@ddn.upes.ac.in; b Inflammation Research Laboratory, Department of Life Science, Parul Institute of Applied Science, Parul University Vadodara Gujarat India; c Department of Biotechnology, Parul Institute of Technology, Parul University Vadodara Gujarat India

## Abstract

This study investigated hydrophobic ion pairing between cromolyn sodium (CS) and cetylpyridinium chloride (CPC), followed by transformation of the resulting CPC–CS complex into nanocarriers using ferulic acid-grafted gelatin (FG) and cinnamic acid-grafted gelatin (CAG) for prolonged drug delivery. The CPC–CS complex was prepared at a 1 : 2 molar ratio and characterized using PXRD, FTIR, ^1^H NMR, polarized light microscopy, and SEM. The resulting FG/CPC–CS and CAG/CPC–CS formulations were evaluated for particle size, drug loading, entrapment efficiency, rheology, drug release, membrane permeation, antioxidant activity, and antibacterial activity. Analytical studies confirmed the formation of a stoichiometric ion pair between the carboxylate groups of CS and pyridinium cations of CPC. FG/CPC–CS and CAG/CPC–CS showed mean hydrodynamic diameters of 301 ± 12 and 237 ± 14 nm, respectively. FG/CPC–CS exhibited higher entrapment efficiency and drug loading than CAG/CPC–CS. Both formulations demonstrated shear-thinning and thixotropic behavior. After 24 h, cumulative drug release was 89.6 ± 0.9% for FG/CPC–CS and 80.8 ± 0.8% for CAG/CPC–CS. FG/CPC–CS also showed greater membrane permeation. Both formulations reduced PMA-induced reactive oxygen species and exhibited antibacterial activity against *E. coli* and *S. aureus*. Aromatic acid-grafted gelatin nanocarriers successfully incorporated the CPC–CS complex and provided sustained drug release, favorable rheological properties, enhanced membrane transport, antioxidant activity, and antibacterial performance. The FG-based formulation showed superior entrapment, release, and permeation, indicating its potential as a prolonged topical delivery platform for cromolyn-based ion-pair systems.

## Abstract 2

Cromolyn sodium: cetylpyridinium chloride ionic complex (CP-CPCS complex) nanocarriers stabilized with ferulic/cinnamic acid-grafted gelatin for enhanced topical delivery.

## Graphical abstract

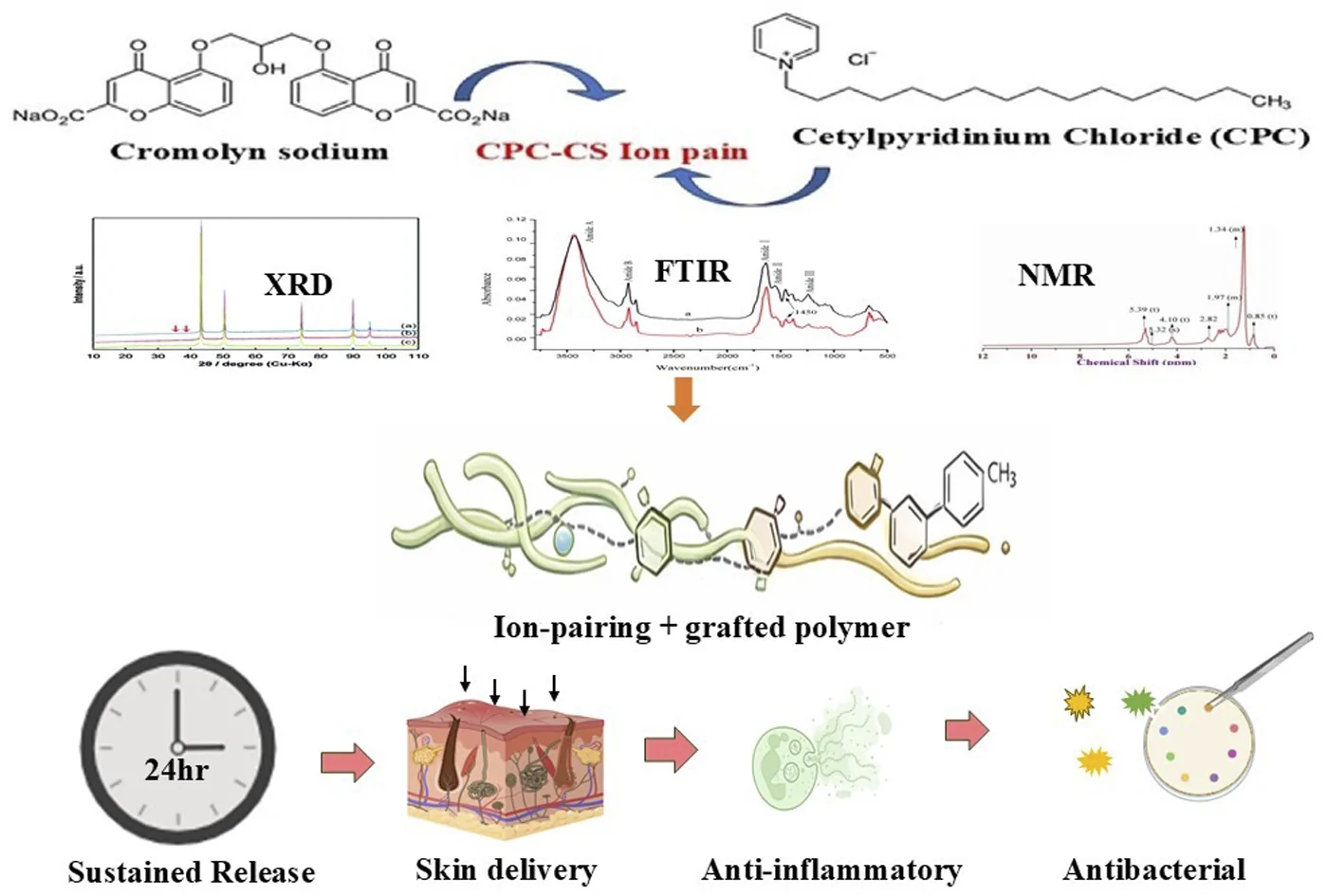

## 1 Introduction

Functional polymeric materials derived from natural macromolecules have attracted significant attention owing to their biocompatibility, chemical tunability, and potential applications in biomedical and pharmaceutical fields.^1,2^ Among these materials, gelatin is frequently used because of its biodegradable polypeptide backbone, compatibility with aqueous media, film-forming ability, and reactive functional groups, including amino, carboxyl, and hydroxyl groups.^3^ These structural characteristics facilitate the chemical modification of gelatin to produce polymeric matrices with improved interfacial, rheological, and drug-retention properties. Modified gelatin-based systems have demonstrated considerable potential for topical and controlled drug delivery, especially when formulation stability, residence time, and controlled release are critical factors. Nevertheless, the release of highly ionic and hydrophilic pharmaceutical compounds from native gelatin matrices is often limited.^4,5^ Drugs with high water solubility can rapidly diffuse through hydrophilic polymer networks, resulting in inadequate retention and reduced sustained-release performance. This limitation is especially relevant to cromolyn sodium (CS), a highly hydrophilic and di-anionic pharmaceutical compound. Owing to its ionic characteristics, CS exhibits high aqueous solubility; however, it shows reduced compatibility with hydrophobic or amphiphilic polymer domains.

Consequently, its incorporation into polymeric delivery systems can result in rapid diffusion, limited molecular retention, and weak interaction with membrane-like biological surfaces. Hydrophobic ion pairing is an effective noncovalent approach for modifying the physicochemical properties of ionic pharmaceutical compounds while maintaining their covalent integrity.^6–8^ This approach relies on electrostatic interactions between oppositely charged species, resulting in the formation of an ion-paired complex with altered solubility, molecular arrangement, and interfacial characteristics. Ion pairing with a cationic amphiphile can reduce the high hydrophilicity of anionic drugs, such as CS, and enhance their compatibility with amphiphilic or hydrophobic polymeric matrices. Cetylpyridinium chloride (CPC) is an amphiphilic cationic surfactant consisting of an aromatic pyridinium headgroup and an extended 16-carbon cetyl chain, possessing a critical micelle concentration (CMC) of approximately 0.90–1.0 mM in aqueous solution at 25 °C.^9^ The thermodynamics of surfactant self-assembly and counterion association are governed by counterion valency, hydration, and polarizability; replacing small, highly hydrated inorganic counterions (Cl^−^) with bulkier, hydrophobic multivalent organic counterions drives spontaneous counterion condensation and ion pairing (Δ*G*° < 0), driven exothermically by electrostatic attraction and entropically by the desolvation of structured hydration water shells surrounding both headgroups and counterions.^9^ Conversely, cromolyn sodium (CS; disodium cromoglycate) is a synthetic bis-chromone derivative containing two ionizable carboxylate groups (–COO^−^) with low p*K*_a_ values (1.5–2.0), rendering it fully dianionic at physiological pH. Owing to its high aqueous solubility (>100 mg mL^−1^) and low octanol–water partition coefficient (log *P* ≈ −1.5), CS suffers from negligible transcellular permeation and rapid physiological clearance. In this context, CPC serves as an ideal cationic pairing amphiphile: electrostatic coordination between the two anionic carboxylate groups of CS and the pyridinium cations of CPC at a 1 : 2 molar ratio effectively neutralizes the formal charges, masks hydrophilicity, and forms a stoichiometric hydrophobic ion pair (CPC)_2_–CS with significantly lowered aqueous solubility and enhanced membrane-interfacial affinity. The formation of these ion pairs may improve the compatibility of CS with modified gelatin matrices and enhance its retention within nanoscale polymeric assemblies. Enhanced regulation of polymer network behavior can be achieved through the grafting of aromatic acids onto gelatin.^10,11^ Grafting aromatic molecules such as ferulic acid and cinnamic acid introduces hydrophobic and aromatic domains into the gelatin backbone. These domains can form hydrogen bonds, hydrophobic associations, and π–π interactions, which influence polymer chain organization, viscosity, interfacial behavior, drug entrapment, and release performance. The phenolic hydroxyl group of ferulic acid-grafted gelatin (FG) is likely to enhance hydrogen bonding and contribute to antioxidant activity. Cinnamic acid-grafted gelatin (CAG) introduces a hydrophobic aromatic structure that may influence network compactness and molecular mobility in distinct ways.

Hydrophobic ion pairing and polymer modification have been studied independently to enhance drug delivery systems;^12^ however, limited attention has been given to the synergistic application of CPC–CS ion pairing with aromatic acid-grafted gelatin matrices. Specifically, the relationships among ion-pair formation, polymer drug interactions, rheological behavior, release profile, permeability, and biological response remain insufficiently understood. A deeper understanding of these structure–property relationships are essential for the development of gelatin-based supramolecular delivery systems for highly ionic pharmaceutical compounds.

In this study, a solvent-mediated ion-exchange technique was used to prepare a CPC–CS ion-paired complex, which was subsequently incorporated into ferulic acid-grafted gelatin and cinnamic acid-grafted gelatin matrices. The physicochemical properties, particle size behavior, drug loading, entrapment efficiency, rheological response, *in vitro* release, *ex vivo* permeability, antioxidant and anti-inflammatory responses, and antibacterial efficacy of the proposed FG/CPC–CS and CAG/CPC–CS formulations were analyzed. The study examined the influence of electrostatic interactions, hydrophobic associations, hydrogen bonding, and aromatic interactions on the performance differences observed between the FG/CPC–CS and CAG/CPC–CS formulations. This study establishes a molecular framework for the development of modified gelatin-based delivery systems aimed at achieving sustained release and improved functional performance of highly ionic pharmaceutical compounds.

## 2 Materials and methods

### 2.1 Materials

Cromolyn sodium (CS; disodium 5,5′-[(2-hydroxypropane-1,3-diyl)bis(oxy)]bis(4-oxo-4*H*-chromene-2-carboxylate); C_23_H_14_Na_2_O_11_, molecular weight: 512.34 g mol^−1^; CAS No. 15826-37-6; purity ≥ 98%) was purchased from Sigma-Aldrich (St. Louis, MO, USA). CS is a synthetic mast cell stabilizer bearing two ionizable carboxylate groups (–COO^−^Na^+^) with p*K*_a_ values of ∼1.5–2.0, exhibiting high aqueous solubility (>100 mg mL^−1^ at 25 °C) and a negative log *P* (−1.5). Cetylpyridinium chloride (CPC; 1-hexadecylpyridinium chloride monohydrate; C_21_H_38_ClN·H_2_O, molecular weight: 358.00 g mol^−1^, purity ≥ 98%, CMC ≈ 0.90–1.0 mM) and gelatin (Type A, from porcine skin, bloom strength ∼175 g) were purchased from Croba Chemie Pvt. Ltd (Mumbai, India) and Spectrochem Pvt. Ltd (Mumbai, India). Dextran, cetylpyridinium chloride (CPC), diphenyleneiodonium chloride (DPI), phorbol 12-myristate 13-acetate (PMA), 2,2-dichlorofluorescein diacetate (DCF-DA) and trypan blue were the ones bought at Spectrochem Pvt. Ltd (Mumbai, India). Density-gradient media of neutrophil isolation (HiSep/GranuloSep) and Hank solution with Balanced Salt Solution (HBSS) and salts that compose it were purchased at HiMedia Laboratories Pvt. Ltd (Mumbai, India). Antimicrobial agar work carried out on MuellerMulligan and HiMedia Laboratories Pvt. Ltd (Mumbai, India) to purchase MuellerMulligan agar, nutrient agar, and nutrient broth. The positive control used in the antibacterial research was Cefixime, which was found as a commercially available source of Indian pharmaceuticals. Amentochlorine-2-1,2-1/2 All chemicals were of analytical grade and were not purified.

### 2.2 Synthesis of cationic cromolyn sodium complex (CPC–CS)

The cationic ion-paired combination of cromolyn sodium (CS) and cetylpyridinium chloride (CPC) was prepared using a solvent-mediated ion-exchange method.^13^ CS and CPC were mixed at a stoichiometric molar ratio of 1 : 2 (CS : CPC) to ensure the electrostatic pairing of both anionic carboxylate groups (–COO^−^) of the dianionic cromolyn with the single quaternary pyridinium cation of two CPC molecules, yielding an electroneutral (CPC)_2_–CS complex. In brief, the required amount of CS was dissolved in distilled water (5 mL) under continuous magnetic stirring until a clear solution was obtained. The pH of the CS solution was adjusted to approximately 7.0 to guarantee complete dissociation of both carboxylic acid moieties (p*K*_a_ ∼ 1.5–2.0) into carboxylate anions (–COO^−^) and induce ion-pair formation. The required amount of CPC was dissolved separately in approximately 100 µL of distilled water to obtain a homogeneous solution.^14^

The CPC solution was slowly added to the CS solution under steady stirring to minimize the formation of local concentration gradients and uncontrolled precipitation. The mixture was stirred for 30–45 min to ensure effective interaction between CS and CPC. The reaction mixture was well-stirred and heated in a water bath at 80–90 °C until a clear and homogeneous solution was obtained. This heating step led to complete solubilization and promoted stoichiometric ion-pair formation between the carboxylate groups of CS and the pyridinium cation of CPC.

The clear solution was then allowed to cool in a refrigerator at 4 °C for 1 h to promote precipitation/crystallization of the CPC–CS ion-paired complex.^15^ The mixture was centrifuged and cooled to collect the resulting precipitate. The precipitate was washed several times with distilled water to remove unreacted CS, CPC, and other water-soluble impurities. Excess water was removed with acetone during the final centrifugation step, and the complex was dried. The CPC–CS precipitate was dried and stored in an airtight vessel for further characterization.^16^

### 2.3 Incorporation of CPC–CS complex into grafted gelatin

Ferulic acid-grafted gelatin (FG) and cinnamic acid-grafted gelatin (CAG), previously synthesized and comprehensively characterized in our earlier investigations,^17,18^ were utilized as amphiphilic polymeric matrices to encapsulate the CPC–CS ion-paired complex. Briefly, in those prior studies, ferulic acid (4-hydroxy-3-methoxycinnamic acid) and *trans*-cinnamic acid were covalently grafted onto the gelatin backbone *via* carbodiimide-mediated amidation (EDC/NHS coupling) between the carboxylic groups of the aromatic acids and the free ε-amino groups of gelatin lysine residues, yielding grafting degrees of approximately 18.4% for FG and 16.2% for CAG. Each formulation was for 100 milligrams of the grafted gelatin mixed with 1.0 mL of distilled water. A magnetic stirrer was further used to stir the solution continuously until the gelatin absorbed the water. The solutions were stirred and fully dissolved after 24 hours at room temperature to make sure that the aqueous polymer solutions remained transparent and unclouded. Five milligrams of the dried CPC–CS were accurately poured in each grafted gelatin solution. The mixtures were stirred constantly until the complex was dissolved completely leading to the formation of a clear solution that was uniform. During this process, the grafted gelatin chains were able to autonomously organize themselves around the hydrophobic ion-paired drug complex through hydrophobic interactions, aromatic π–π stacking, and electrostatic stabilization, thereby forming drug-loaded polymeric nanoparticles *in situ*. The formulations were transparent, meaning that CPC–CS was properly encompassed to the grafted gelatin masses at the molecular level. The optical translucency of the dispersions, together with their nanoscale size as evidenced by dynamic light scattering, suggested the presence of colloidally stable nanocarriers rather than molecular solutions. They were then used to characterize, release and test antibacterial activity.

### 2.4 Drug loading and entrapment efficiency

The entrapment efficiency (EE, %) and drug loading (DL, %) of the CPC–CS complex in the aromatic acid-grafted gelatin nanoparticles were determined by separating and estimating the quantity of free, unentrapped drug. Briefly, the nanoparticle dispersion was centrifuged at 8000 rpm (6708×*g*) for 25 min at 4 °C using a refrigerated centrifuge, and the supernatant was carefully collected. Using UV-visible spectrophotometry at *λ*_max_ = 326 nm, the concentration of free cromolyn sodium in the supernatant was determined. The following equations were used to calculate the entrapment efficiency and drug loading:

1 Entrapment efficiency (EE, %) = [(*W*_total drug_ − *W*_free drug_)/*W*_total drug_] × 100

2 Drug loading (DL, %) = [(*W*_total drug_ − *W*_free drug_)/*W*_total nanoparticles_] × 100

where *W*_total drug_ is the total weight of cromolyn sodium initially added to the formulation, *W*_free drug_ is the weight of unentrapped cromolyn sodium quantified in the supernatant, and *W*_total nanoparticles_ is the total dry weight of the recovered nanoparticles. Each experiment was carried out in triplicate (*n* = 3), and values are expressed as mean ± standard deviation.

### 2.5 Characterization

#### 2.5.1 X-ray diffraction (XRD), Fourier transform infrared (FTIR), and ^1^H nuclear magnetic resonance (^1^H NMR) analyses

The crystalline properties of cetylpyridinium chloride (CPC); cromolyn sodium (CS) and CPC–CS were studied using powder X-ray diffraction. The XRD was measured using Bruker D8 Advance ECO diffractometer at 40 kV and 30 mA using Cu K-radiation (1.5406 A), which collects a total of 30 measurements.^19^ Each sample was carefully ground but first accurately weighed and then the required sample ground was placed on the sample holder to achieve a well packed and flat surface of powder to be analyzed. The FTIR spectroscopy was used to examine CPC–CS, as well as the intermediates formed by the grafting reactions, when they are esterified.^20^ As a spectrometer, a PerkinElmer Frontier FT-IR ATR was used. All samples were finely ground into a fine powder then the attenuated total reflectance (ATR) mode was used to analyses the sample in the solid form. Fourth, spectra were measured between 4000 to 400 cm^−1^ over the range with a resolution of 0.5 cm^−1^. Each spectrum was measured 32 times on average in order to promote the signal-to-noise ratio. The spectra obtained were used to identify those vibrations that were specific to functional groups, subtract shifts related to interactions between ions, and assay the presence of grafting in esterification of the derivatives of gelatin. Peak places and differences in the intensity allowed analyzing the interactions of molecules and changes in the structure within the CPC–CS and polymer matrix.

Proton nuclear magnetic resonance (^1^H NMR) spectroscopy was performed on a Bruker Avance III HD 500 MHz spectrometer at 298 K to elucidate molecular-level interactions and chemical shift perturbations. Accurately weighed samples (15 mg) of pure CPC, pure CS, and the synthesized CPC–CS complex were completely dissolved in 0.6 mL of deuterated dimethyl sulfoxide (DMSO-d_6_, 99.8 atom % *D*; Sigma-Aldrich). The spectra were acquired using conventional acquisition parameters and chemical shifts (*δ*) were expressed in parts per million (ppm) in relation to the peak of the solvent.^21^

#### 2.5.2 Polarized light microscopy (PLM) and SEM analysis

The microstructural organization and birefringence of cetylpyridinium chloride (CPC) and cromolyn sodium (CS) and their CPC–CS were studied with the help of polarized light microscopy.^22^ The sample (finely powdered) was approximately 1 mg of each that was evenly spread on a glass microscope slide and pressed lightly to form a thin, uniform layer that was observable by the naked eye. Micrographic analysis was done by using a trinocular polarizing microscope that had cross-polarized illumination. The images were taken with 20× and 50× magnifications to view crystalline domains, the pattern of extinction, and morphological changes on the basis of the formation of ion-pairs.

The electron microscopy (SEM) was used to study the surface markings of cetylpyridinium chloride (CPC), cromolyn sodium (CS), and the ion-paired complex (CPC–CS). About 1 mg of samples each was carefully placed on conductive carbon tape on aluminum SEM stubs. Since the samples were composed of nonconductive organic matter, a sputter coater (SEM-C mode) was used to coat the samples to a thin layer with gold–palladium (Au–Pd) to reduce the effect of charging on imaging. A low-vacuum, low-force scanning electron microscope (SEM-LV-LF) from the Analytical Instruments Facility at H. N. B. Garhwal University was used to examine the microstructures.^23^

#### 2.5.3 Interfacial tension, particle size analysis, and rheological characterization

The interfacial tension of the cromolyn sodium solutions was measured with the help of the KRUSs Drop Shape Analyzer (DSA25, KRUSS GmbH, Germany) utilizing a pendant drop method.^24^ A set of CS solutions was prepared with its concentration of 0–20 mg mL^−1^ in the distilled water. At the tip of a stainless-steel needle, droplets were produced under the controlled environment using a needle. The shape of the pendant drop was measured by a high-resolution camera and the Young–Laplace equation was utilized in the calculation of the interfacial tension automatically. Three times was added to each concentration to provide some level of consistency. The values obtained as the result were used to measure interfacial behavior of cromolyn sodium depending on the concentration and its relevance to the ion-pairing interactions. The particle size distribution was checked by means of Dynamic Light Scattering (DLS), which was conducted using Malvern Zetasizer Nano Series (Model: ZEN 1690, Type S). Flited water was applied to make diluted preparations that had the best scattering intensity. Probe sonicate was applied to the measurements, with and without aggregation to assess stability of agglomeration. The sonicated samples were run in the pulsed interval of 10 seconds on and 10 seconds off with a run time of 3 minutes. This method reduced the number of thermal effects during the sonication.^25^ Passage of all tests was done in triplicates and average hydrodynamic diameter was given.

A rotational rheometer (Anton Paar MCR 92, Austria) equipped with a concentric cylinder (cup-and-bob, CC27) measuring geometry and an automated Peltier temperature control system (accuracy ±0.05 °C) was used to investigate the rheological properties and flow behavior of the formulations.^26^ Steady-state flow curves and apparent dynamic viscosity (*η*) were determined across a shear rate range of *γ̇* = 0.1 to 100 s^−1^ at three temperatures: 25 °C (ambient storage), 37 °C (physiological surface temperature), and 50 °C (elevated thermal stress). The shear stress (*τ*) was recorded as a function of shear rate (*γ̇*), and apparent viscosity was calculated directly *via η* = *τ*/*γ̇*. Flow curves were modeled mathematically using the Power-law (Ostwald-de Waele) model: *τ* = *K*·*γ̇*^*n*^ (where *K* is the consistency index in Pa s^*n*^, and *n* is the dimensionless flow behavior index) and the Herschel–Bulkley model: *τ* = *τ*_0_ + *K*_HB_·*γ̇*^*n*^_HB_ (where *τ*_0_ is the yield stress in Pa). Thixotropic hysteresis was quantified from continuous linear shear rate ramp cycles (0.1 → 100 → 0.1 s^−1^) by integrating the area between the upward and downward flow curves (*A*_hyst_ in Pa s s^−1^), indicating the mechanical work required for structural breakdown and network recovery kinetics.

### 2.6 Drug release and permeation studies

#### 2.6.1 *In vitro* drug release

The profile of release of cromolyn sodium on the CPC–CS-loaded nanoparticle formulations was tested using a USP Type II dissolution apparatus *in vitro*. The diffusion barrier is a dialysis membrane, which was pre-activated by placing it in the hot sodium bicarbonate solution of 15 minutes then rinsed in large quantities of the double-distilled water. Five milligrams (CPC–CS complex) of cromolyn sodium added to the hydrated membrane and firmly sealed in a 900 mL of phosphate-buffered saline (PBS, pH 7.4) at 37 °C were placed under conditions of 37 ± 0.5 °C.^26^ The rate of paddle rotation was adjusted to 100 rpm so as to have a constant level of mixing in the process. 5 mL of aliquots were taken at predetermined time points (0, 1, 2, 3, 4, 5, 6, 8, 10, 12, 16 and 24 h) and immediately exchanged with fresh PBS to keep sink conditions constant. The amount of absorbance of cromolyn sodium at a wavelength, 326 nm, max of 1 was determined using a UV-visible spectrophotometer (UV-1900, Shimadzu, Japan). The release rate of drugs was obtained in a cumulative way and different kinetic models were utilized to examine the mechanism of drug release.^27^ These were the zero-order (cumulative release *versus* time) and first-order (log cumulative drug remaining *versus* time) and Higuchi (cumulative release *versus* square root of time), Korsmeyer–Peppas (log cumulative release *versus* log time), and the Hixson–Crowell (square root of remaining drug *versus* time) models. The optimal fit to describe the process of release kinetics and diffusion process was modelled based on the highest regression coefficient (*R*^2^).^28^

#### 2.6.2 *Ex vivo* skin permeation

*Ex vivo* studies involving dialysis were done using a dialysis membrane with molecular weight of 10–12 kDa. The clay was pre-treated in the immersion of the membrane in hot sodium bicarbonate solution of 15 minutes and then thoroughly rinsed in the double-distilled water. The permeation tests were done in the Franz diffusion cell. This is done by ensuring that the active membrane was carefully placed between the receptor and donor compartments (Sahu *et al.*, 2026). The CPC–CS was incorporated into the membrane surface consistently and the PBS (pH 7.4) was placed in the chamber holding the receptors and constantly stirred. To ensure that sink conditions were observed, 2 mL samples were removed out of receptor medium at different intervals (0, 1, 2, 3, 4, 5, and 6 h) and replaced by the equal volume of fresh PBS. Triage of every sample was conducted thrice at 326 nm to determine the quantity of permeated cromolyn sodium. The total drug permeation with time was calculated using the effective membrane surface area. The steady-state flux (2 µg cm^−2^ h^−1^) and the permeability coefficient^29^ were obtained using the linear part of the permeation profile.

### 2.7 Antimicrobial activity and cellular assays

#### 2.7.1 Isolation of neutrophils

Healthy volunteers had their blood taken as the periphery samples by way of consent in accordance with the ethical approval (PUIECHR/PIMSR/00/081734/7502) and consent. The neutrophils were isolated through dextran sedimentation then density-gradient centrifugation.^30^ Whole blood was then centrifuged at 2000 rpm and 10 minutes at room temperature so as to separate the plasma. After that, dextran was used to sediment the buffy coat in 1 percent dextran in 30 seconds. Supernatant with high concentration of leukocyte was thoroughly overlaid on a Histopaque 1119/1077 gradient and centrifuged at 700 g during 15 minutes. The bottom part, with neutrophils was rinsed twice in Hanks Balanced Salt Solution (HBSS: 138 mM NaCl, 2.7 mM KCl, 8.1 mM Na_2_HPO_4_, 1.5 mM K_2_HPO_4_, 10 mM glucose) and mixed once more in 2 mL of HBSS. Cell viability was proven using trypan blue exclusion.

#### 2.7.2 Assessing the levels of reactive oxygen species (ROS) within cellular environments

The reactive oxygen species (ROS) formation within human neutrophils was measured using 2,7-dichlorofluorescein diacetate (DCF-DA).^31^ Neutrophils (1 × 10^6^ cells) had been pretreated at 37 °C, 5 minutes with either (FG/CPC–CS, 10 µg mL^−1^) or (CAG/CPC–CS, 10 µg mL^−1^). After the treatment, the cells were kept in the dark (with DCF-DA, 100-1) during 5 minutes. Phorbol 12-myristate 13-acetate (PMA, 10 nM) was then used to induce the oxidative stress. Fluorescence intensity relating to production of intracellular ROS was measured using a multimode fluorescence reader (excitation: 488 nm; emission: 520 nm). A comparison of the fluorescence was made against the control cells that were not stimulated and was provided as the mean stimulation index and standard deviation (SD).

#### 2.7.3 Antimicrobial susceptibility testing (well diffusion method)

The analysis of antimicrobial susceptibility is done through a method called well diffusion. The agar well diffusion procedure was employed to determine the bactericidal effect of the formulations, FG/CPC–CS and CAG/CPC–CS. *Escherichia coli* (ATCC 25922) and *Staphylococcus aureus* (ATCC 25923) standard strains were reactivated on nutrient agar at 37 °C and grown in nutrient broth until the mid-log. Bacterium suspensions were brought to 0.5 McFarland standard (approximately 1.5 × 10 8 CFU mL^−1^). Mueller–Hinton agar (MHA) was prepared and autoclaved then cooled to 45 °C and added into sterile Petri plates to the depth of 4 mm. Bacterial suspension was evenly whitened on agar base. A sterile coryu76k borer was used to prepare 6 mm-diameter wells, and then 10 3 L each test formulation was pipetted into the wells. Pre-incubation of the plates was done at 37 °C along with 20 to 40 minutes incubation and then the plates were incubated overnight at the same temperature. The antibacterial efficacy was measured in millimeters as the zone of inhibition (ZOI).

## 3 Results and discussion

### 3.1 Preparation of the CPC–CS and its incorporation into grafted gelatin

A clear, homogeneous solution was obtained from the CPC–CS system through controlled mixing and thermal treatment, indicating a consistent interaction between cromolyn sodium and cetylpyridinium chloride during the ion-exchange process. Gradual cooling and storage at 4 °C resulted in the formation of a white precipitate. This precipitation indicated a reduction in aqueous solubility following the association of CS with CPC, likely due to charge neutralization and the influence of CPC's hydrophobic cetyl chain.^32^

The recovered CPC–CS product was a solid material, in contrast to the original aqueous CS solution, thereby indicating the formation of an associated ion-paired material. However, precipitation alone is not sufficient to confirm complete complex formation; therefore, the product was characterized using XRD, FTIR, and ^1^H NMR to analyze changes in crystallinity, the functional group environment, and molecular-level interactions. The developed FG/CPC–CS and CAG/CPC–CS formulations exhibited visual homogeneity, showing no signs of turbidity or phase separation after incorporation into ferulic acid-grafted gelatin and cinnamic acid-grafted gelatin matrices. This indicated that the CPC–CS product was compatible with the grafted gelatin matrices and could be dispersed within the polymeric network. The stability of the formulations may be attributed to the interplay of electrostatic, hydrophobic, hydrogen-bonding, and aromatic interactions between CPC–CS and the grafted gelatin chains.

### 3.2 Characterization

The studies of PXRD, FTIR, and ^1^H NMR revealed that the molecular structure and physical state were profoundly modified upon ion-pair complex formation between cromolyn sodium (CS) and cetylpyridinium chloride (CPC). Powder X-ray diffraction (PXRD) patterns of pure CPC, pure CS, and the synthesized CPC–CS complex are depicted in Fig. 1a. Pure CPC exhibited intense, sharp Bragg reflections at low diffraction angles (2*θ* = 6.8°, 13.5°, 20.3°, and 24.1°), indicative of a highly ordered, layered crystalline lattice characteristic of long-chain alkyl pyridinium salts. Pure CS displayed prominent crystalline reflections at 2*θ* = 5.4°, 10.8°, 16.2°, 22.0°, and 26.5°, reflecting the hydrated crystalline packing of disodium cromoglycate. In contrast, the PXRD diffractogram of the CPC–CS complex displayed a markedly altered diffraction profile (Fig. 1a). The intense, sharp diffraction reflections characteristic of crystalline CPC and CS were virtually extinguished or drastically attenuated. Instead, the complex showed a broad, diffuse amorphous background halo superimposed with several weak, broadened reflections at 2*θ* ≈ 7.2°, 18.5°, and 21.4° (shown magnified in the inset of Fig. 1a). The substantial reduction in diffraction intensity of the complex is attributable to the steric hindrance and conformational disruption imposed by the interdigitating C16 hydrocarbon chains around the rigid cromolyn core, which hinders long-range three-dimensional crystal lattice packing. Rather than representing a simple physical mixture (which would retain superimposable sharp peaks of both precursors), these diffraction changes provide convincing evidence for non-covalent stoichiometric ion pairing and the emergence of an intimate supramolecular mesomorphic/semi-crystalline phase with reorganized intermolecular packing.^17^ The FTIR spectra of pure CPC, pure CS, and the CPC–CS complex further corroborated the formation of the ion pair through distinct functional group vibrational shifts (Fig. 1b). For pure cromolyn sodium, the spectrum displayed characteristic bands associated with its carboxylate and chromone scaffold: an asymmetric carboxylate stretching vibration (*ν*_as_(COO^−^)) at 1622 cm^−1^, a symmetric carboxylate stretching vibration (*ν*_s_(COO^−^)) at 1405 cm^−1^, a chromone ring carbonyl stretching vibration (*ν*(C=O)) at 1640 cm^−1^, aromatic ring skeletal C=C vibrations at 1572 and 1475 cm^−1^, and ether C–O–C stretching vibrations at 1098 and 1264 cm^−1^. The broad absorption band centered at 3380–3420 cm^−1^ corresponds to O–H stretching from the secondary alcoholic hydroxyl group of the central spacer as well as lattice-associated hydration water. Pure CPC exhibited strong asymmetric and symmetric aliphatic C–H stretching vibrations from its hexadecyl hydrocarbon tail at 2918 and 2850 cm^−1^, respectively, along with pyridinium ring C=N^+^ and C=C vibrations at 1635, 1486, and 1466 cm^−1^, and characteristic pyridinium C–H bending vibrations at 778 and 682 cm^−1^. Importantly, the moderately broad band observed in the pure CPC spectrum around 3334 cm^−1^ does not arise from an N–H stretching vibration (as CPC is a fully alkylated quaternary pyridinium compound possessing no N–H bonds); rather, it is definitively assigned to the O–H stretching vibration of hydration/adsorbed water molecules associated with the hygroscopic quaternary chloride salt in commercial cetylpyridinium chloride monohydrate (C_21_H_38_ClN·H_2_O). In the FTIR spectrum of the CPC–CS complex (Fig. 1b), prominent vibrational perturbations confirmed strong electrostatic ion pairing: the asymmetric carboxylate band shifted from 1622 cm^−1^ to 1608 cm^−1^, and the symmetric carboxylate band shifted from 1405 cm^−1^ to 1388 cm^−1^ with marked peak broadening and intensity attenuation. Concurrently, the pyridinium ring vibrations of CPC shifted from 1486 cm^−1^ to 1472 cm^−1^, while the intense aliphatic C–H stretching bands of the cetyl chain (2918 and 2850 cm^−1^) were fully retained in the complex. The simultaneous preservation of the hydrophobic cetyl tail vibrations and significant bathochromic shifts in the carboxylate and pyridinium absorptions unequivocally confirm stoichiometric electrostatic ion-pair formation between the anionic carboxylate groups of CS and the cationic pyridinium nitrogen of CPC, without covalent modification.^33^

**Fig. 1:**
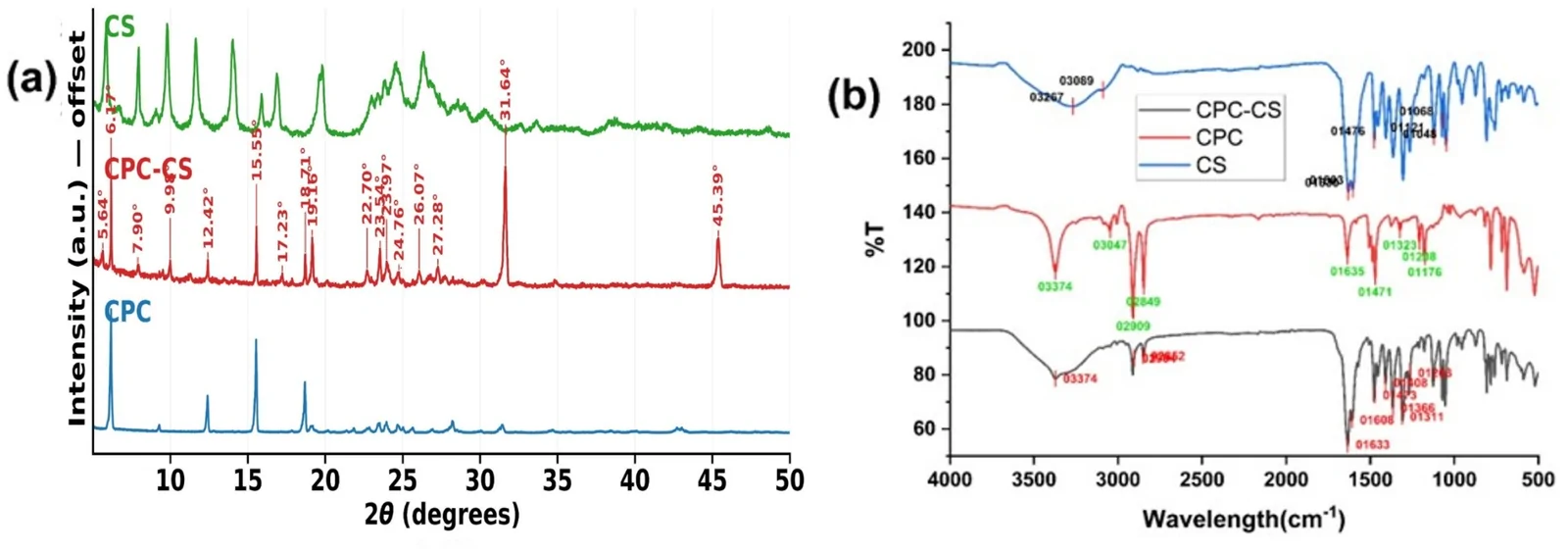
Solid-state crystallographic and vibrational spectroscopic characterization of ion-pair formation. (a) Powder X-ray diffraction (PXRD) patterns of pure cetylpyridinium chloride (CPC), pure cromolyn sodium (CS), and the CPC–CS ion-pair complex recorded with Cu Kα radiation (*λ* = 1.5406 Å) over 2*θ* = 5.0–50.0° (step size 0.019°), showing the extinction of precursor Bragg reflections and the appearance of broadened reflections in the complex (magnified in the inset); (b) attenuated total reflectance Fourier-transform infrared (ATR-FTIR) spectra of CPC, CS, and the CPC–CS complex over 4000–400 cm^−1^ (32 scans, resolution 0.5 cm^−1^), illustrating bathochromic shifts in carboxylate stretching vibrations (*ν*_as_(COO^−^) at 1622 → 1608 cm^−1^; *ν*_s_(COO^−^) at 1405 → 1388 cm^−1^) and pyridinium ring vibrations, alongside retention of aliphatic cetyl C–H bands (2918 and 2850 cm^−1^).

High-resolution ^1^H NMR spectroscopy (500 MHz, DMSO-d_6_) provided unambiguous molecular-level confirmation of stoichiometric ion-pair complexation (Fig. 2). The residual solvent quintet of DMSO-d_6_ at *δ* = 2.50 ppm and the residual water singlet at *δ* = 3.33 ppm were identified and calibrated as internal references. For pure cromolyn sodium (Fig. 2b), complete proton assignments and integrations confirmed its dianionic disodium salt structure: chromone aromatic protons appeared at *δ* = 7.74 ppm (t, *J* = 8.4 Hz, 2H, H-7, H-7′), *δ* = 7.15 ppm (d, *J* = 8.4 Hz, 2H, H-8, H-8′), and *δ* = 6.95 ppm (d, *J* = 8.4 Hz, 2H, H-6, H-6′), while the chromone olefinic protons resonated at *δ* = 6.60 ppm (s, 2H, H-3, H-3′). The central glyceryl spacer protons were resolved at *δ* = 4.35–4.45 ppm (m, 4H, –O–CH_2_–), *δ* = 4.18 ppm (m, 1H, –CH(OH)–), and *δ* = 5.65 ppm (br d, 1H, secondary aliphatic –OH). Because CS is present as a disodium carboxylate salt (–COO^−^Na^+^), no carboxylic acid proton is observed. In pure CPC (Fig. 2a), the aromatic pyridinium protons were clearly assigned to *δ* = 9.12 ppm (d, *J* = 5.6 Hz, 2H, α-pyridinium), *δ* = 8.65 ppm (t, *J* = 7.8 Hz, 1H, γ-pyridinium), and *δ* = 8.19 ppm (t, *J* = 7.0 Hz, 2H, β-pyridinium). The aliphatic cetyl protons were assigned as follows: –N^+^–CH_2_– at *δ* = 4.62 ppm (t, *J* = 7.5 Hz, 2H), –N^+^–CH_2_–CH_2_– at *δ* = 1.93 ppm (m, 2H), –(CH_2_)_13_– bulk methylene envelope at *δ* = 1.18–1.35 ppm (m, 26H), and the terminal –CH_3_ triplet at *δ* = 0.85 ppm (t, *J* = 6.8 Hz, 3H). Total proton integration (38H) matched the molecular composition of CPC perfectly. In the ^1^H NMR spectrum of the CPC–CS complex (Fig. 2c), substantial chemical shift perturbations and peak broadening were observed. The α-pyridinium protons of CPC shifted upfield from *δ* = 9.12 ppm to *δ* = 8.97 ppm (Δ*δ* = −0.15 ppm), and the –N^+^–CH_2_– protons shifted from *δ* = 4.62 ppm to *δ* = 4.54 ppm (Δ*δ* = −0.08 ppm), driven by the magnetic shielding effect exerted by the electron-rich aromatic chromone ring and carboxylate oxygens of CS in close spatial proximity. Simultaneously, the cromolyn aromatic signals exhibited peak broadening and slight upfield shifts. Regarding the aliphatic cetyl protons of CPC in the complex, the resonances in the region of *δ* = 1.18–1.35 ppm exhibited noticeable line broadening (transverse relaxation *T*_2_ shortening) and attenuated peak height. This phenomenon arises because the two hydrophobic C16 hydrocarbon chains of the paired (CPC)_2_–CS complex undergo spontaneous intermolecular hydrophobic association/micro-domain clustering in polar DMSO, restricting local rotational mobility and causing peak broadening into a diffuse resonance band. These spectroscopic findings confirm stoichiometric 1 : 2 (CS : CPC) ion-pair complexation mediated by electrostatic attraction between the carboxylate groups of CS and the pyridinium headgroups of CPC, complemented by hydrophobic packing of the cetyl chains.

**Fig. 2:**
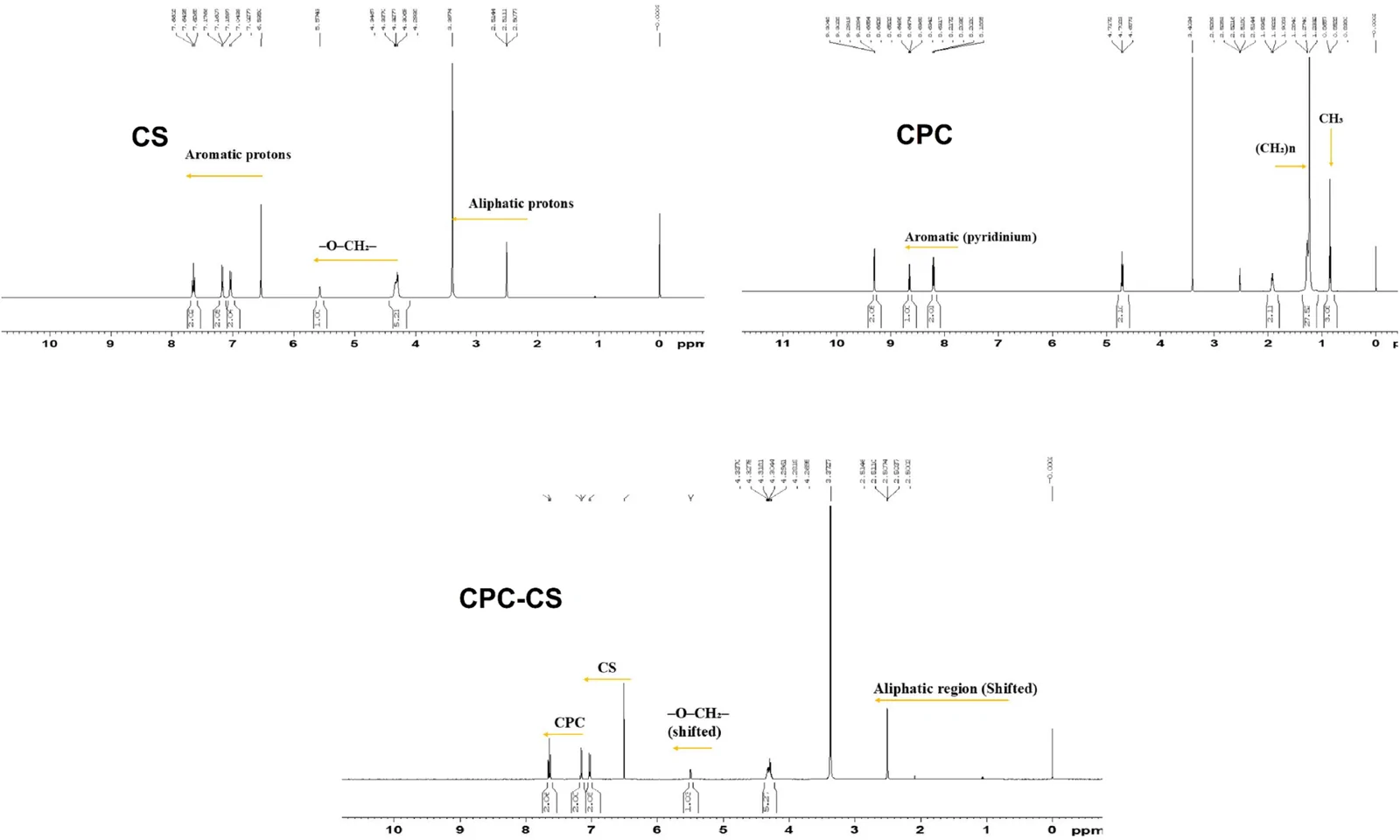
500 MHz ^1^H NMR spectra illustrating stoichiometric ion-pair complex formation recorded in DMSO-*d*_6_ at 298 K. Cetylpyridinium chloride (CPC) displaying pyridinium aromatic protons (*δ* = 8.19–9.12 ppm) and cetyl chain aliphatic protons (*δ* = 0.85–4.62 ppm); cromolyn sodium (CS) showing chromone aromatic protons (*δ* = 6.95–7.74 ppm), olefinic protons (*δ* = 6.60 ppm), and glyceryl linker protons (*δ* = 4.18–5.65 ppm); CPC–CS ion-paired complex demonstrating upfield shifts of α-pyridinium protons (Δ*δ* = −0.15 ppm) due to aromatic ring shielding, downfield shifts of CS protons, and characteristic line broadening of the C16 aliphatic envelope resulting from hydrophobic clustering. Residual solvent peaks are calibrated at *δ* = 2.50 ppm (DMSO) and *δ* = 3.33 ppm (H_2_O).

As described previously, cromolyn sodium is a divalent dicarboxylic acid derivative composed of two planar chromone rings joined by a 2-hydroxypropane-1,3-dioxy flexible linker. At physiological and neutral pH, both carboxylate groups (–COO^−^) are fully ionized. Complexation occurs at a strict 2 : 1 stoichiometric ratio (CPC : CS), wherein two monovalent cetylpyridinium cations associate with the two terminal carboxylate groups of a single cromolyn dianion *via* strong electrostatic ion-pair coordination (–COO^−^⋯^+^N-py). This ionic pairing neutralizes the polar charges of the drug, while the two flexible hexadecyl (C16) tails orient cooperatively in parallel, stabilized by intermolecular van der Waals and hydrophobic interactions. When the (CPC)_2_–CS complex is incorporated into aromatic acid-grafted gelatins, multiple complementary non-covalent forces may drive nanoparticle formation: (i) the aromatic rings of ferulic acid and cinnamic acid can engage in extensive π–π stacking with both the electron-deficient pyridinium headgroups of CPC and the planar chromone cores of CS; (ii) the hydrophobic cetyl tails interdigitate into the hydrophobic microdomains created by the grafted aromatic moieties; and (iii) in the case of ferulic acid-grafted gelatin (FG), the free phenolic hydroxyl group (–OH) and *ortho*-methoxy group (–OCH_3_) act as potent hydrogen-bond donors and acceptors, establishing directed multivalent hydrogen-bonding networks with the ether linkages and carbonyl oxygens of CS and the gelatin polypeptide backbone. This additional hydrogen-bonding capacity in FG accounts for its superior entrapment efficiency, enhanced structural stability, and higher physiological viscosity compared to CAG, which relies exclusively on unsubstituted phenyl π–π stacking and hydrophobic interactions.

### 3.3 Polarized light microscopy (PLM) and SEM analysis

Polarized light microscopy (PLM) and scanning electron microscopy (SEM) were utilized to characterize the morphology, microstructural organization, and solid-state transitions of CPC, CS, and the CPC–CS complex (Fig. 3). PLM micrographs revealed pronounced differences in crystalline birefringence (Fig. 3a). Pure CPC exhibited highly birefringent, elongated needle-like crystal platelets with sharp extinction angles and vibrant interference colors (Fig. 3a(1)), characteristic of the ordered crystal lattice of quaternary alkylpyridinium halides, with typical crystal lengths of 25–60 µm and widths of 2–5 µm. Pure cromolyn sodium displayed distinct microcrystalline tabular plates with moderate, localized birefringence and defined edges (Fig. 3a(2)), possessing dimensions of 15–40 µm, consistent with its known hydrated crystalline morphology. Upon stoichiometric ion-pair formation, the solid CPC–CS complex displayed compact, cohesive supramolecular agglomerates with substantially attenuated, diffuse birefringence and suppressed interference colors (Fig. 3a(3)), forming cohesive domains of 10–25 µm. This marked reduction in optical anisotropy confirms the loss of individual precursor crystal lattices and the emergence of an intimate supramolecular ion-pair phase, in full agreement with the PXRD results. When dispersed in aqueous medium, the CPC–CS complex spontaneously reorganized into finely dispersed colloidal micro-aggregates (<5 µm) displaying minimal residual birefringence (Fig. 3a(4)), indicating excellent aqueous dispersibility and compatibility with polymeric matrices. SEM micrographs corroborated these structural observations at high magnification (Fig. 3b). Pure CPC exhibited elongated, slender prismatic rods and crystalline needle bundles (lengths 10–35 µm, widths 1.5–4 µm) with smooth crystalline faces (Fig. 3b(1)). Pure CS exhibited distinct block-like crystalline plates and flat polygonal sheets (lengths 15–45 µm, thicknesses 1–3 µm) with clean fracture facets (Fig. 3b(2)). In stark contrast, the CPC–CS complex revealed a completely transformed, unified surface architecture characterized by cohesive, interwoven lamellar sheets and wrinkled multi-layered flakes (lateral dimensions 5–18 µm, sheet thickness ∼150–350 nm) with evident folding and fusion between layers (Fig. 3b(3)). This distinct lamellar texture results from strong electrostatic neutralization between the carboxylate moieties of CS and the pyridinium headgroups of CPC, accompanied by parallel interdigitation of the hydrophobic cetyl tails. The complete disappearance of the individual precursor rod and block morphologies into cohesive lamellar sheets unequivocally demonstrates that CPC–CS is a true supramolecular ion-pair complex rather than a physical mixture.

**Fig. 3:**
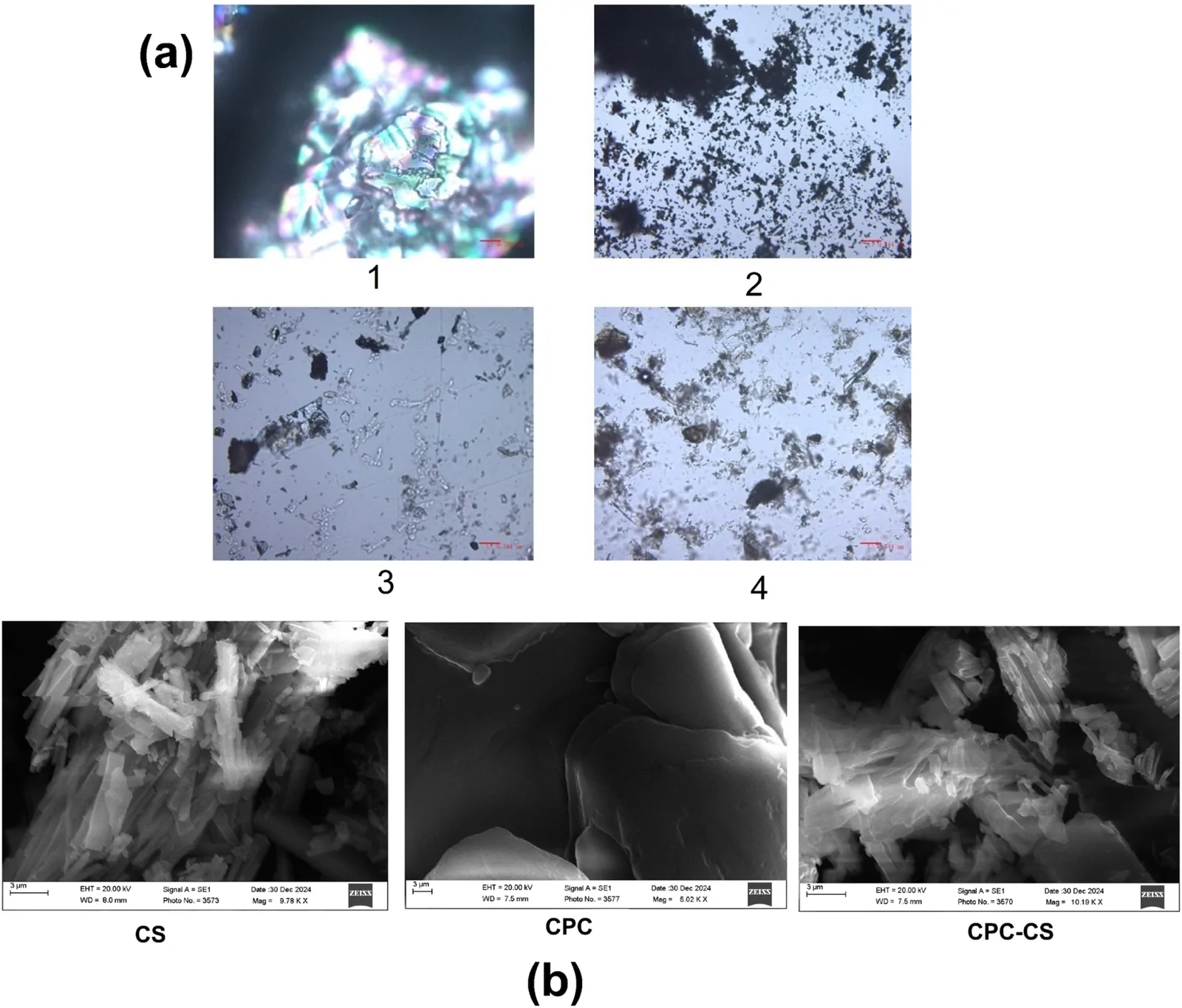
Morphological and microstructural evaluation of precursors and the CPC–CS ion pair. (a) Polarized light microscopy (PLM) micrographs showcasing birefringence patterns and optical anisotropy: (1) pure CPC exhibiting highly birefringent crystalline needles (25–60 µm × 2–5 µm); (2) pure CS displaying moderate birefringence on tabular microcrystalline plates (15–40 µm); (3) solid CPC–CS complex displaying attenuated, diffuse birefringence on cohesive supramolecular agglomerates (10–25 µm); (4) CPC–CS aqueous dispersion showing fine colloidal micro-aggregates (<5 µm); (b) scanning electron microscopy (SEM) micrographs illustrating surface morphology: (1) pure CPC showing elongated prismatic rods and needle bundles (10–35 µm × 1.5–4 µm); (2) pure CS displaying smooth block-like crystalline plates (15–45 µm × 1–3 µm); (3) CPC–CS complex revealing cohesive, interwoven lamellar sheets and wrinkled multi-layered flakes (5–18 µm, thickness ∼150–350 nm).

This increases its solubility and compatibility in further formulation into the grafted gelatin matrices and explains the reduction of the particle size after the sonication process and its sensitivity to shear in the rheological experiment.^34^

### 3.4 Interfacial tension studies, particle size analysis and rheological studies

The interfacial, colloidal, and rheological characteristics of CPC–CS-loaded grafted gelatin formulations were analyzed to assess their physicochemical stability, flow behavior, and suitability for topical delivery applications.^35^ The incorporation of CPC–CS into the grafted gelatin matrices influenced the interfacial and structural characteristics of the formulations, which may be attributed to the synergistic effects of ionic, hydrophobic, hydrogen-bonding, and aromatic interactions (Fig. 4–7 and Tables 1–3).^36^

**Fig. 4:**
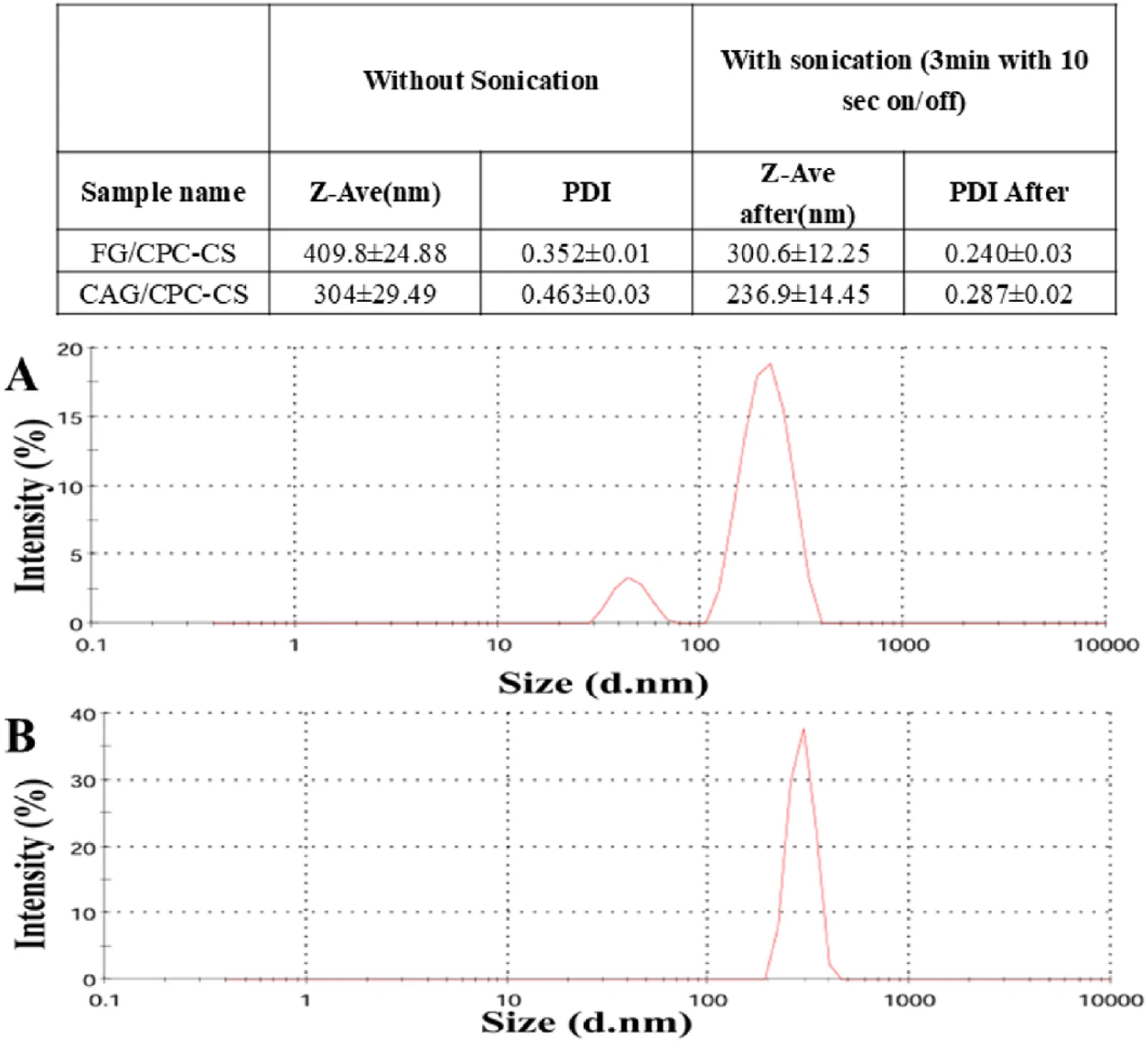
Dynamic light scattering (DLS) hydrodynamic size distribution profiles and effect of probe sonication on colloidal uniformity. Size distribution curves of (A) FG/CPC–CS and (B) CAG/CPC–CS before and after pulsed probe sonication (10 s on/10 s off, 3 min duration, 25 °C), with axes formatted according to IUPAC standards as ‘Intensity/%’ *versus* ‘Size/nm’, demonstrating monomodal nanoscale distribution and reduction of secondary agglomerates.

**Fig. 5:**
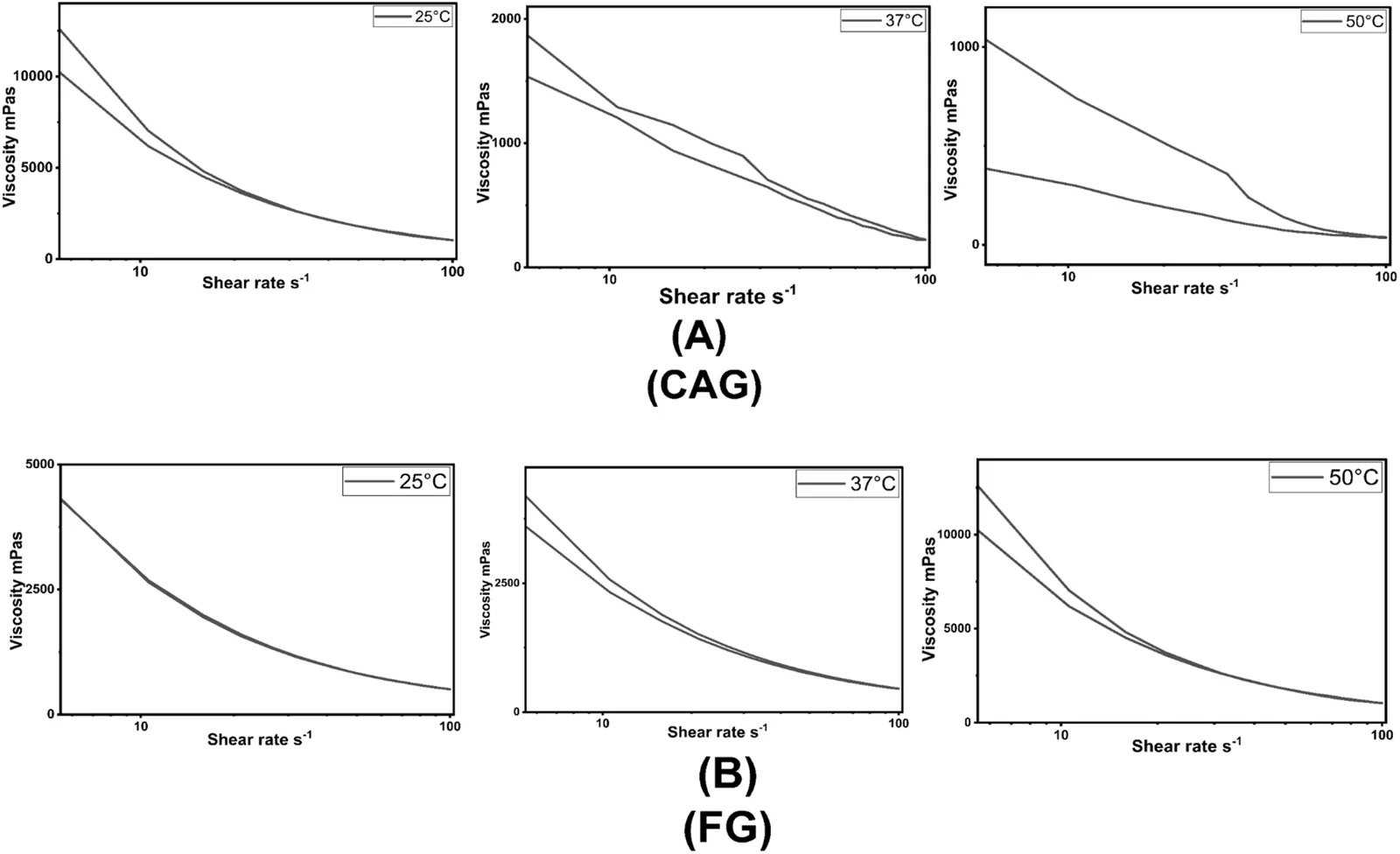
Continuous linear ramp rheological flow curves of grafted gelatin nanoparticle systems. Dynamic viscosity as a function of shear rate across *γ̇* = 0.1 to 100 s^−1^ at 25 °C, 37 °C, and 50 °C for (A) cinnamic acid-grafted gelatin (CAG/CPC–CS) and (B) ferulic acid-grafted gelatin (FG/CPC–CS), illustrating pseudoplastic flow and superior thermal resilience of the ferulic acid matrix. Axes formatted as ‘Viscosity/(Pa s)’ *versus* ‘Shear rate/s^−1^’.

**Fig. 6:**
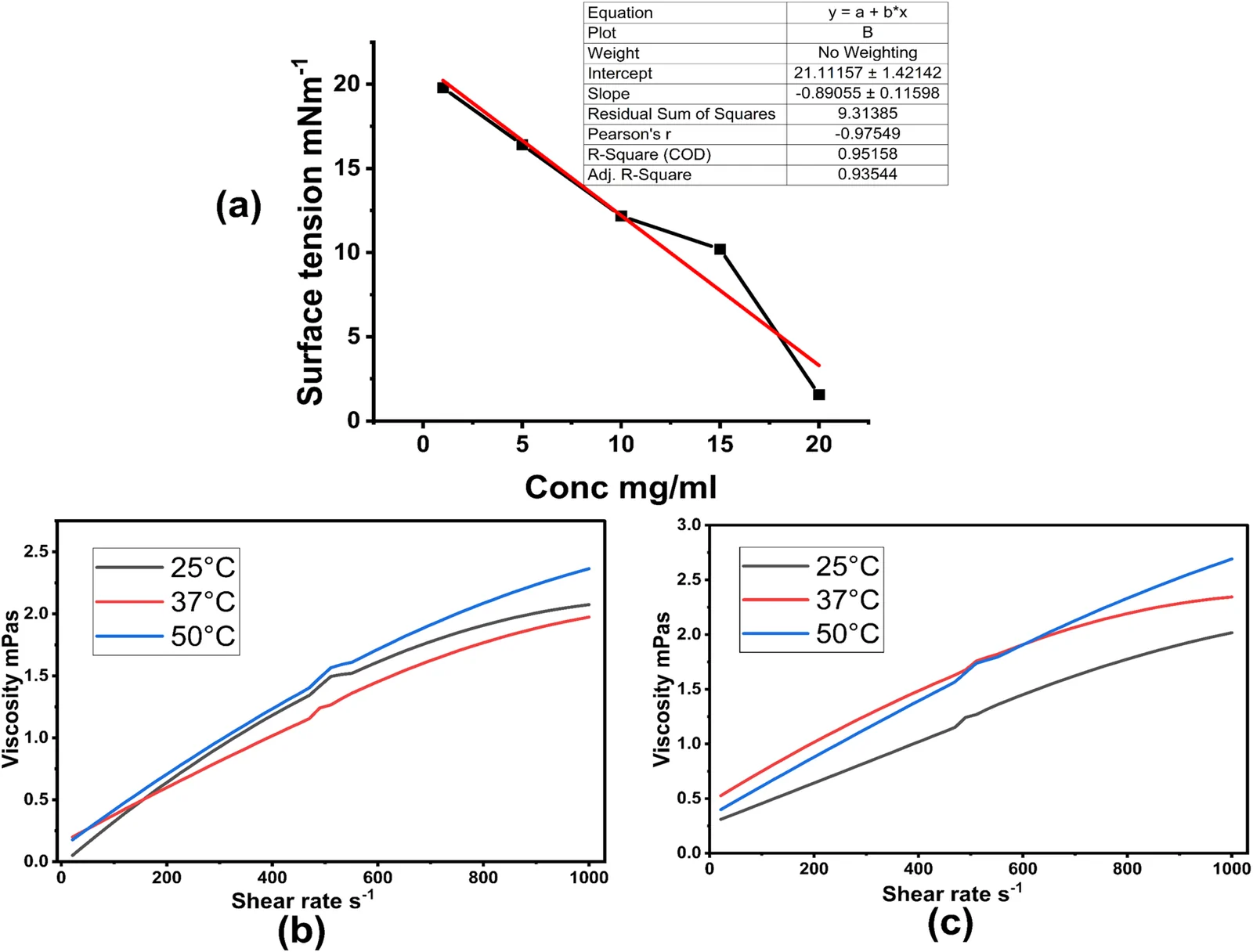
Interfacial surface tension and steady-state shear flow behavior. (a) Surface tension of cromolyn sodium solutions (0–20 mg mL^−1^) measured *via* the pendant drop method at 25 °C, showing reduction of interfacial energy; (b) steady-state viscosity *versus* shear rate (0.1–100 s^−1^) of CAG/CPC–CS at 25 °C, 37 °C, and 50 °C; (c) steady-state viscosity *versus* shear rate of FG/CPC–CS under identical conditions, illustrating robust pseudoplastic behavior. All axes formatted as ‘Quantity/unit’.

**Fig. 7:**
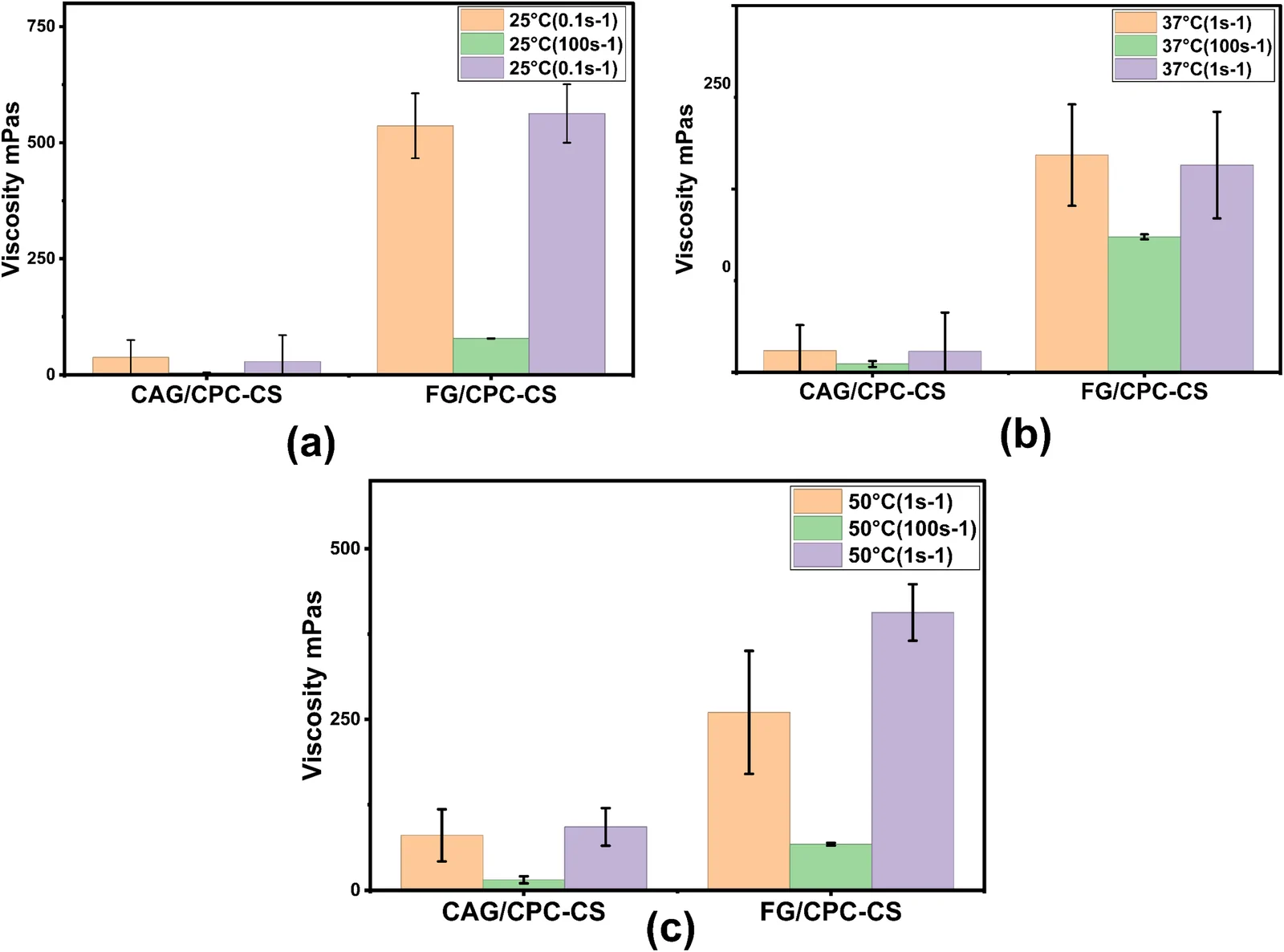
Comparative dynamic viscosity analysis of FG/CPC–CS and CAG/CPC–CS under low and high shear conditions. Dynamic viscosity values at (a) 25 °C, (b) 37 °C, and (c) 50 °C under low shear (0.1 s^−1^) and high shear (100 s^−1^) rates, demonstrating the superior viscosity retention of FG/CPC–CS at physiological temperature (37 °C) due to phenolic hydrogen bonding.

**Table 1:** Drug loading and entrapment efficiency of CPC–CS in aromatic acid-grafted gelatin nanoparticle formulations (mean ± SD, *n* = 3)

Formulation	Entrapment efficiency/%	Drug loading/%
FG/CPC–CS	83.6 ± 2.1	4.18 ± 0.12
CAG/CPC–CS	76.4 ± 1.8	3.82 ± 0.09

**Table 2:** True viscosity and hysteresis loop area of CPC–CS formulations at different temperatures

Formulation	Temperature/°C	True viscosity at 100 s^−1^/(Pa s)	Hysteresis area/(Pa s s^−1^)
FG/CPC–CS	25	1.0352	6.7437
FG/CPC–CS	37	0.4521	1.2075
FG/CPC–CS	50	0.5044	2.3026
CAG/CPC–CS	25	1.0422	1.2075
CAG/CPC–CS	37	0.2228	3.5849
CAG/CPC–CS	50	0.0375	1.1394

**Table 3:** Power-law and Herschel–Bulkley model fitting parameters for CPC–CS formulations

Formulation	Temperature/°C	Power-law *K*/(Pa s^*n*^)	Power-law *n*	*R* ^2^ (PL)	Herschel–Bulkley *τ*_0_/Pa	Herschel–Bulkley *K*/(Pa s^*n*^)	Herschel–Bulkley *n*	*R* ^2^ (HB)
FG/CPC–CS	25	2.9294	0.2344	0.9353	0.000	2.9294	0.2344	0.9353
FG/CPC–CS	37	8.7714	0.2476	0.8829	0.000	8.7714	0.2476	0.8829
FG/CPC–CS	50	3.2886	0.2435	0.9112	0.000	3.2886	0.2435	0.9112
CAG/CPC–CS	25	1.4826	0.2476	0.9024	0.000	1.4826	0.2476	0.9024
CAG/CPC–CS	37	1.2327	0.3471	0.9008	1.9385	1.1959	0.3586	0.9089
CAG/CPC–CS	50	0.2907	0.3548	0.8270	0.1416	0.2893	0.3576	0.8270

Interfacial tension analysis indicated a reduction in surface energy after the integration of CPC–CS, suggesting the amphiphilic characteristics of the ion-paired complex. The cationic cetylpyridinium group is likely to orient at the interface, while the cromolyn sodium moiety remains associated with the aqueous phase. This interfacial arrangement enhances wetting, spreading, and interaction of the formulation with biological surfaces. This behavior is critical in topical delivery systems, where uniform distribution and close proximity to the membrane surface are vital for optimal drug release and penetration.^17^

Dynamic light scattering (DLS) analysis confirmed the formation of nanoscale dispersed systems in both the FG/CPC–CS and CAG/CPC–CS formulations (Fig. 4 and Table 1). The mean hydrodynamic diameter (*Z*-average) of FG/CPC–CS was 301 ± 12 nm (polydispersity index, PDI = 0.28 ± 0.02), which exceeded that of CAG/CPC–CS at 237 ± 14 nm (PDI = 0.24 ± 0.02), indicating stronger intermolecular interactions and greater hydration swelling within the ferulic acid-grafted gelatin matrix.^37^ This may be attributed to the presence of phenolic hydroxyl groups in ferulic acid, which could enhance hydrogen bonding together with hydrophobic and aromatic interactions. In all cases, controlled probe sonication (pulsed 10 s on/10 s off for 3 min) led to reductions in particle size to 248 ± 10 nm for FG/CPC–CS and 192 ± 9 nm for CAG/CPC–CS, with improved monodispersity (PDI < 0.20), indicating the disintegration of loose secondary aggregates and the achievement of colloidal uniformity (Fig. 4). The decrease in particle size following sonication suggested that the supramolecular structures were reversible and could be reorganized without fully destabilizing the system. In addition to particle size distribution, the assessment of drug loading and encapsulation efficiency provided further validation of the compatibility of CPC–CS with the grafted gelatin matrices. The entrapment efficiency of FG/CPC–CS (83.6 ± 2.1%) was significantly higher than that of CAG/CPC–CS (76.4 ± 1.8%; *p* < 0.05), accompanied by superior drug loading (4.18 ± 0.12% *vs.* 3.82 ± 0.09%), suggesting stronger interactions between CPC–CS and the ferulic acid-grafted gelatin network. The inherent adaptability of the FG structure may make it better suited for accommodating the hydrophobic ion-paired complex within the polymeric matrix. Conversely, the comparatively more hydrophobic and compact structure of CAG may restrict the loading capacity for CPC–CS.^33,38^

The enhanced entrapment efficiency of FG/CPC–CS suggests a more favorable microenvironment within the ferulic acid-grafted gelatin matrix for retaining the CPC–CS complex. This may be attributed to the combined influence of hydrogen bonding, aromatic interactions, and hydrophobic interactions between the CPC–CS complex and the grafted gelatin chains. The reduction in the amount of free drug in the supernatant further suggests that CPC–CS was effectively encapsulated within the nanoscale polymeric assemblies.^39^

A comprehensive rheological investigation was conducted to assess the flow behavior, shear responsiveness, and thermal stability of the CPC–CS-loaded grafted gelatin formulations (Fig. 5–7 and Tables 2, 3). Both FG/CPC–CS and CAG/CPC–CS exhibited pronounced non-Newtonian, pseudoplastic (shear-thinning) flow characteristics across all investigated temperatures (25 °C, 37 °C, and 50 °C). Dynamic apparent viscosity (calculated *via η* = *τ*/*γ̇*) decreased monotonically with increasing shear rate (*γ̇* = 0.1 to 100 s^−1^). At low shear rates (*γ̇* → 0), both systems demonstrated elevated zero-shear apparent viscosity, reflecting an unperturbed, three-dimensional physical network established through chain entanglement, hydrophobic cetyl chain clustering, and aromatic π–π stacking. Under applied shear, hydrodynamic forces progressively align the polymeric chains and disentangle the colloidal nanoaggregates along the flow streamlines, disrupting weak physical crosslinks and reducing flow resistance. This shear-thinning behavior is highly advantageous for topical delivery, ensuring effortless spreading during application (high shear) while preserving high viscosity at rest (low shear) to prevent formulation drainage.^40^

Additionally, temperature-dependent viscosity profiles indicated that the internal structures of both formulations were sensitive to thermal conditions (Fig. 5 and Table 2). At 25 °C, high viscosity was observed for both formulations, attributable to enhanced physical crosslinking and restricted chain mobility. As temperature increased to 37 °C and 50 °C, viscosity declined as a result of thermal softening within the polymeric structure and partial dissociation of intermolecular physical junctions. However, the differences between FG/CPC–CS and CAG/CPC–CS indicated that the chemical identity of the aromatic graft had a profound impact on the thermomechanical stability of the formulations (Fig. 5 and 7).

The step shear-rate analysis provided additional evidence of the shear-responsive nature of the formulations. Both systems exhibited viscosity transitions from low to high shear rates and *vice versa*, suggesting reversible structural collapse and partial recovery. FG/CPC–CS exhibited greater viscosity retention under shear than CAG/CPC–CS, indicating a more robust interconnected polymer–drug network.^41,42^ This effect may result from enhanced hydrogen bonding and aromatic interactions within the ferulic acid-grafted gelatin matrix. Conversely, CAG/CPC–CS exhibited a more fluid response, suggesting reduced shear resistance and faster structural relaxation.^18^

The quantitative rheological parameters presented in Table 2 and Fig. 7 further confirmed the distinct thermomechanical flow behavior of the formulations. At 25 °C, the dynamic viscosity values of FG/CPC–CS and CAG/CPC–CS at 100 s^−1^ were comparable, measured at 1.0352 and 1.0422 Pa s, respectively, indicating well-developed physical networks under ambient conditions. When the temperature was raised to skin surface temperature (37 °C), CAG/CPC–CS experienced a sharp 78.6% drop in viscosity to 0.2228 Pa s, and collapsed further to 0.0375 Pa s at 50 °C due to rapid thermal chain relaxation. In sharp contrast, FG/CPC–CS demonstrated exceptional thermo-rheological stability, maintaining an apparent viscosity of 0.4521 Pa s at 37 °C and 0.5044 Pa s at 50 °C (Table 2 and Fig. 7). This remarkable thermal tolerance arises from the free phenolic hydroxyl groups of ferulic acid, which form thermally resilient, directed hydrogen-bonding networks with the cromolyn ether/carbonyl oxygens and gelatin amide backbones, effectively resisting thermal dissociation.^36^

The thixotropic hysteresis loop area (*A*_hyst_) presented in Table 2 directly reflects the energy required to break down the internal supramolecular network under shear. At 25 °C, FG/CPC–CS exhibited a large hysteresis area (6.7437 Pa s s^−1^), indicating an extensive entangled network. At physiological temperature (37 °C), the hysteresis area of FG/CPC–CS decreased to 1.2075 Pa s s^−1^, indicating rapid and reversible structural recovery following shear-induced breakdown. In contrast, CAG/CPC–CS displayed an increased hysteresis area at 37 °C (3.5849 Pa s s^−1^), suggesting delayed structural recovery and more extensive irreversible network deformation under physiological shear forces.^43^

The mathematical model-fitting parameters presented in Table 3 provide quantitative verification of the non-Newtonian flow behavior. The Power-law flow behavior index values (*n*) for all samples were well below 1.0 (ranging between 0.23 and 0.35, *R*^2^ > 0.88), definitively confirming strong shear-thinning (pseudoplastic) properties. The consistency index (*K*), which quantifies network strength, was markedly higher for FG/CPC–CS at 37 °C (*K* = 8.7714 Pa s^*n*^) than for CAG/CPC–CS (*K* = 1.2327 Pa s^*n*^), demonstrating superior network cohesion in the presence of ferulic acid grafts. Furthermore, Herschel–Bulkley model fitting revealed negligible yield stress (*τ*_0_ = 0.000 Pa) for FG/CPC–CS across all temperatures, indicating unhindered, smooth flow initiation upon shear application. Conversely, CAG/CPC–CS exhibited a finite yield stress at 37 °C (*τ*_0_ = 1.9385 Pa) and 50 °C (*τ*_0_ = 0.1416 Pa), indicating the presence of a weak, fragile internal yield threshold prone to mechanical breakdown.^37^

Overall, the interfacial, particle size, entrapment, and rheological results indicate that the incorporation of CPC–CS significantly influenced the properties of the grafted gelatin matrices. FG/CPC–CS exhibited enhanced structural stability and shear responsiveness, which could be advantageous for prolonged residence and sustained-release characteristics. In contrast, CAG/CPC–CS showed comparatively lower viscosity at physiological and elevated temperatures, indicating enhanced flow and spreading capabilities. Consequently, the rheological findings align with the particle size and entrapment data, indicating that molecular interactions between CPC–CS and grafted gelatin play a crucial role in formulation stability, flow characteristics, and potential topical applicability^44^

The shear-responsive structure derived from FG/CPC–CS may contribute to controlled drug diffusion and improved formulation retention in topical applications.^45^ This rheological behavior is directly relevant to the sustained-release and permeation performance discussed in Section 6.5.

### 3.5 *In vitro* release, release kinetics, and *ex vivo* permeation studies

The release profile of cromolyn sodium in the CPC–CS-loaded gelatin-based formulations (FG/CPC–CS and CAG/CPC–CS) was studied *in vitro* by use of a study employed the dialysis membrane technique after 24 hours in phosphate buffer saline (PBS, pH 7.4). Fig. 8a demonstrates the cumulative release curves of both formulations, and the corresponding kinetic modeling parameters are summarized in Table 4.

**Fig. 8:**
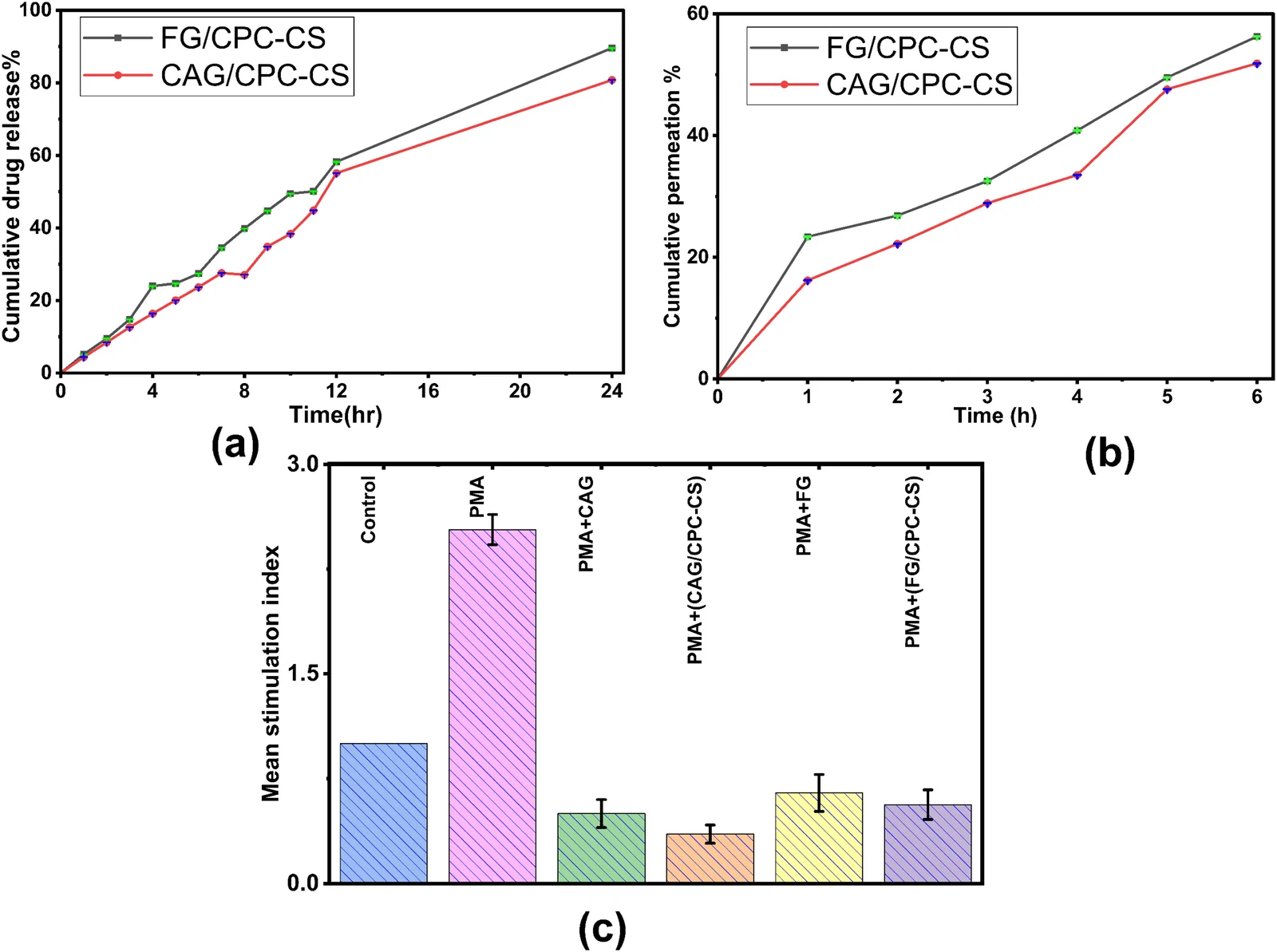
Investigation of *in vitro* drug release, *ex vivo* permeation, and intracellular antioxidant activity of CPC–CS-loaded grafted gelatin formulations. (a) Cumulative *in vitro* release profiles of cromolyn sodium from FG/CPC–CS and CAG/CPC–CS in PBS (pH 7.4, 37 ± 0.5 °C, 100 rpm) over 24 h (mean ± SD, *n* = 3); (b) cumulative *ex vivo* permeation of cromolyn sodium across a dialysis membrane utilizing Franz diffusion cells (effective area 1.77 cm^2^, receptor volume 12 mL, 37 °C) over 6 h; (c) inhibitory effect of FG, CAG, and their cromolyn sodium-loaded formulations on PMA-induced intracellular reactive oxygen species (ROS) generation in human neutrophils, quantified *via* DCF-DA fluorescence (excitation 488 nm, emission 520 nm) and expressed as mean stimulation index ± SD (*n* = 3; *p < 0.05 *vs.* PMA control). All axes formatted as ‘Quantity/unit’.

**Table 4:** Kinetic modeling of cromolyn sodium release from FG/CPC–CS and CAG/CPC–CS formulations (PBS, pH 7.4)

Kinetic model	FG/CPC–CS *R*^2^	FG/CPC–CS *k*	CAG/CPC–CS *R*^2^	CAG/CPC–CS *k*
Zero order	0.9653	3.8743	0.9702	3.5115
First order	0.9635	0.0914	0.9654	0.0683
Higuchi	0.9463	19.4880	0.9098	17.2750
Korsmeyer–Peppas	0.9879	0.0548	0.9912	0.0443
Hixson–Crowell	0.9931	0.1020	0.9807	0.0828

In the course of the trial, both FG/CPC–CS and CAG/CPC–CS formulations were found to release the medication controlled and constant.^46^ The gradual release period was reported as 6 to 8 hours after which, the continuous and the extended-release period took place through the rest of the 24-hour period. After 24 h, cumulative drug release reached 89.6 ± 0.9% for FG/CPC–CS and 80.8 ± 0.8% for CAG/CPC–CS. The drug-polymer interaction in the CAG matrix was more effective compared to the interaction of the drug and polymer in the FG matrix.

This increased liberation of FG/CPC–CS can be accounted by the increased hydrophiliability of the ferulic acid grafted gelatin, and the flexibility of its polymeric backbone. This aids in the diffusion of CPC–CS ion pair in the medium of dissolution. Conversely, the markedly decreased release rate of CAG/CPC–CS can be attributed to stronger hydrophobic forces created through grafting of cinnamic acid to form a more compact polymerized structure that is impeding to movement of drugs. This release mechanism was explained by comparing the dissolution information based on the use of different kinetic models which include zero-order, first-order, Higuchi, Korsmeyer–Peppas and Hixson–Crowell models. Regression coefficients (*r*^2^) and the constant kinetics (*k*) of the regression are summarised in Table 4. Both expressions yielded the highest correlation coefficients as shown by the Korsmeyer–Peppas model with the values of *r*^2^ FG/CPC–CS being 0.9879 and CAG/CPC–CS being 0.9912. It means that the most suitable model to describe the drug release mechanism is the Korsmeyer–Peppas model. The zero-order model was quite linear implying that the rate of release was relatively constant especially in the case of FG/CPC–CS. According to Korsmeyer–Peppas model, the release exponent (*n*) of drug (FG/CPC–CS and CAG/CPC–CS) was 0.55 and 0.61 (0.43–0.85), respectively, which implies that the drug release process of both formulations had non-Fickian diffusion profile. The results suggest that the release of cromolyn sodium can be controlled using grafted gelatin ones because swelling of the polymer can be controlled and the ionic interactions of CPC–CS and the polymer backbone can be regulated. This makes them useful as delivery vehicles of topical administration that is sustainable. To determine the transdermal diffusion of cromolyn sodium of FG/CPC–CS and CAG/CPC–CS formulations in an *ex vivo* environment, a Franz diffusion cell was used during 6 hours(Fig. 8b).^47^ Both formulations demonstrated sustained permeation across the membrane. After 6 h, cumulative permeation reached 56 ± 2% for FG/CPC–CS and 52 ± 2% for CAG/CPC–CS. FG/CPC–CS achieved a higher steady-state flux (*J* = 0.046 ± 0.003 µg cm^−2^ h^−1^) than CAG/CPC–CS (*J* = 0.041 ± 0.002 µg cm^−2^ h^−1^), indicating superior membrane transport efficiency. Also evidenced by the associated permeability coefficients was that the transfer between FG/CPC–CS was the best. Amphiphilic nature of CPC–CS, as well as flexible polymeric structure of ferulic acid-grafted gelatin explain the enhanced permeation characteristics of FG/CPC–CS that enable the interaction and diffusion of drugs to membrane lipids. Conversely, it is possible that the reduction of the higher penetration of CAG/CPC–CS can be associated with the existence of more hydrophobic interactions in the cinnamic acid-modified matrix resulting in a lower movement of drugs.^48^

### 3.6 Antimicrobial activity and cellular assays

DFG-DA test was used to measure the effects of FG, CAG as well as their cromolyn sodium-loaded formulations (CAG/CPC–CS and FG/CPC–CS) on reactive oxygen species (ROS) generation in human neutrophils. Compared to the control cells that were not stimulated, PMA stimulation greatly increased the process of ROS generation. This proves that there was an effective induction of oxidative stress. CAG and FG treatment also showed significant reduction in the level of ROS induced by PMA showing their own antioxidant properties. The addition of cromolyn sodium to the polymeric matrix also minimized the production of ROS and it was found to be statistically significant. CAG/CPC–CS and FG/CPC–CS were found to have better antioxidant activity than their polymer controls had Table 5 (Fig. 8c). The effect of FG/CPC–CS on decreasing the level of ROS was the most pronounced in this case (Fig. 4). The composite formulations have a greater antioxidant activity because of the synergistic effect of cromolyn sodium and the grafted gelatin matrices that lead to a performance of a stronger nature. Cromolyn sodium works by stabilizing the inflammatory cells and inhibiting the emission of the mediators. The carriers are probably polymeric and increase cellular absorption and cellular retention of the drug. Ferulic acid of FG/CPC–CS adds more phenolic groups which are capable of trapping the free radicals and thus more effect of the antioxidant is augmented.

**Table 5:** Effect of FG, CAG, and their cromolyn sodium-loaded formulations on PMA-induced intracellular ROS generation in human neutrophils (mean ± SD, *n* = 3)

Treatment formulation	Mean stimulation index	SD (±)
CAG	0.50	0.10
CAG/CPC–CS	0.35	0.07
FG	0.65	0.13
FG/CPC–CS	0.56	0.11

The antibacterial activity of the formulations against *Escherichia coli* (ATCC 25922) and *Staphylococcus aureus* (ATCC 25923) was evaluated using the agar well diffusion assay method. While unformulated polymer controls (CAG and FG) demonstrated weak baseline antibacterial activity (zones of inhibition, ZOI = 6.5 ± 0.5 mm and 7.2 ± 0.4 mm, respectively) and pure cromolyn sodium exhibited no significant intrinsic bactericidal action (ZOI < 7.0 mm), the composite formulations exhibited pronounced, broad-spectrum antibacterial efficacy. CAG/CPC–CS produced clear inhibition zones of 15.6 ± 0.7 mm against *E. coli* and 17.8 ± 0.8 mm against *S. aureus*. Significantly, the strongest antibacterial activity was achieved with FG/CPC–CS, yielding inhibition zones of 18.4 ± 0.8 mm against *E. coli* and 21.2 ± 0.9 mm against *S. aureus*, approaching the efficacy of the positive control Cefixime (24.5 ± 1.0 mm). The potent bactericidal activity of the composite formulations is predominantly conferred by the cationic pyridinium moiety of cetylpyridinium chloride (CPC), which interacts electrostatically with the negatively charged bacterial peptidoglycan and lipopolysaccharide cell wall components, inducing membrane destabilization, leakage of cytoplasmic contents, and rapid bacterial cell death. The superior antibacterial performance of FG/CPC–CS reflects its enhanced membrane affinity and sustained release of the active ion pair.

Optimized membrane interaction and increased residence are the reasons why FG/CPC–CS has an improved cellular antioxidant and antibacterial activity as it was evidenced in *ex vivo* permeation and rheological stability tests.^49^

## 4 Conclusions

In conclusion, this study demonstrates a rational supramolecular strategy for delivering the highly hydrophilic, dianionic drug cromolyn sodium *via* stoichiometric hydrophobic ion pairing with cetylpyridinium chloride and subsequent nanoparticle stablization with aromatic acid-grafted gelatin. Stoichiometric 1 : 2 (CS : CPC) ion-pair complexation was driven by electrostatic coordination between the two ionized carboxylate groups (–COO^−^) of cromolyn and the quaternary pyridinium headgroups of two CPC molecules, complemented by parallel hydrophobic interdigitation of the hexadecyl (C16) hydrocarbon tails. This non-covalent modification masked the dianionic charges of the drug, dramatically suppressed aqueous solubility, and transformed the crystalline lattice of the precursors into an intimate, cohesive lamellar supramolecular mesophase, as confirmed by PXRD, FTIR, ^1^H NMR, PLM, and SEM. Incorporation of the (CPC)_2_–CS complex into ferulic acid-grafted gelatin (FG) and cinnamic acid-grafted gelatin (CAG) yielded colloidally stable nanoparticles with mean hydrodynamic diameters of 301 ± 12 nm and 237 ± 14 nm, respectively. The presence of phenolic hydroxyl groups in FG established a cooperative multivalent network of hydrogen bonding and aromatic π–π stacking with the complex, resulting in superior drug entrapment efficiency (83.6 ± 2.1%), enhanced thermal and rheological stability at physiological temperature (37 °C), and favorable shear-thinning thixotropy. These structural attributes translated into sustained, non-Fickian drug release over 24 h, enhanced *ex vivo* membrane permeation, potent suppression of PMA-induced neutrophil reactive oxygen species (ROS), and broad antibacterial activity against *E. coli* and *S. aureus*. Overall, these findings provide comprehensive molecular and physicochemical insights into ion-pair-mediated self-assembly and establish aromatic acid-grafted gelatin nanoparticles as a promising multifunctional platform for the sustained topical delivery of ionic therapeutic agents.

## Ethical statement

This study involved no *in vivo* animal studies and there were no vertebrate or invertebrate animals used in this study. 3. Cellular Experiments Human neutrophils were isolated from peripheral blood samples obtained from healthy volunteers after informed consent and approval of Institutional Ethics Committee (Approval number. PUIECHR/PIMSR/00/081734/7502). Anti\microbial activities were carried out on the standard strains of *Escherichia coli* (ATCC 25922) and *Staphylococcus aureus* (ATCC 25923). No animal or human skin was excised, instead permeation research was conducted in a Franz diffusion cell using a synthetic dialysis membrane.

## Author contributions

Sukirti Dobriyal contributed to writing the original draft, investigation, methodology, data curation, validation, and visualization. Sarvjeet Das was involved in investigation, methodology, and validation. Juhi Saxena contributed to investigation and data curation. Anupam Jyoti was responsible for formal analysis, validation, and visualization. Prashant Shukla contributed to conceptualization, supervision, methodology, formal analysis, writing – review and editing, and validation.

## Conflicts of interest

The authors declare that they have no known competing financial interests or personal relationships that could have appeared to influence the work reported in this paper.

## Supplementary Material

RA-OLF-D6RA07879F-s001

RA-OLF-D6RA07879F-s002

RA-OLF-D6RA07879F-s003

RA-OLF-D6RA07879F-s004

## Data Availability

The data supporting this article have been included in the article and as part of the supplementary information (SI). Supplementary information is available. See DOI: https://doi.org/10.1039/d6ra07879f.
