# Supplementary material for: Dual antimicrobial and anti-inflammatory efficacy of cetylpyridinium–cromolyn ion-pair nanoparticles delivered via modified gelatin
Source: RSC Adv. 2026 Nov 4. Online ahead of print. doi: 10.1039/d6ra07879f (PMC13732725; doi:10.1039/d6ra07879f)
Supplement: Supplementary file 1 [file RA-OLF-D6RA07879F-s002.pdf]

Supplementary Information (SI) for RSC Advances.
This journal is © The Royal Society of Chemistry

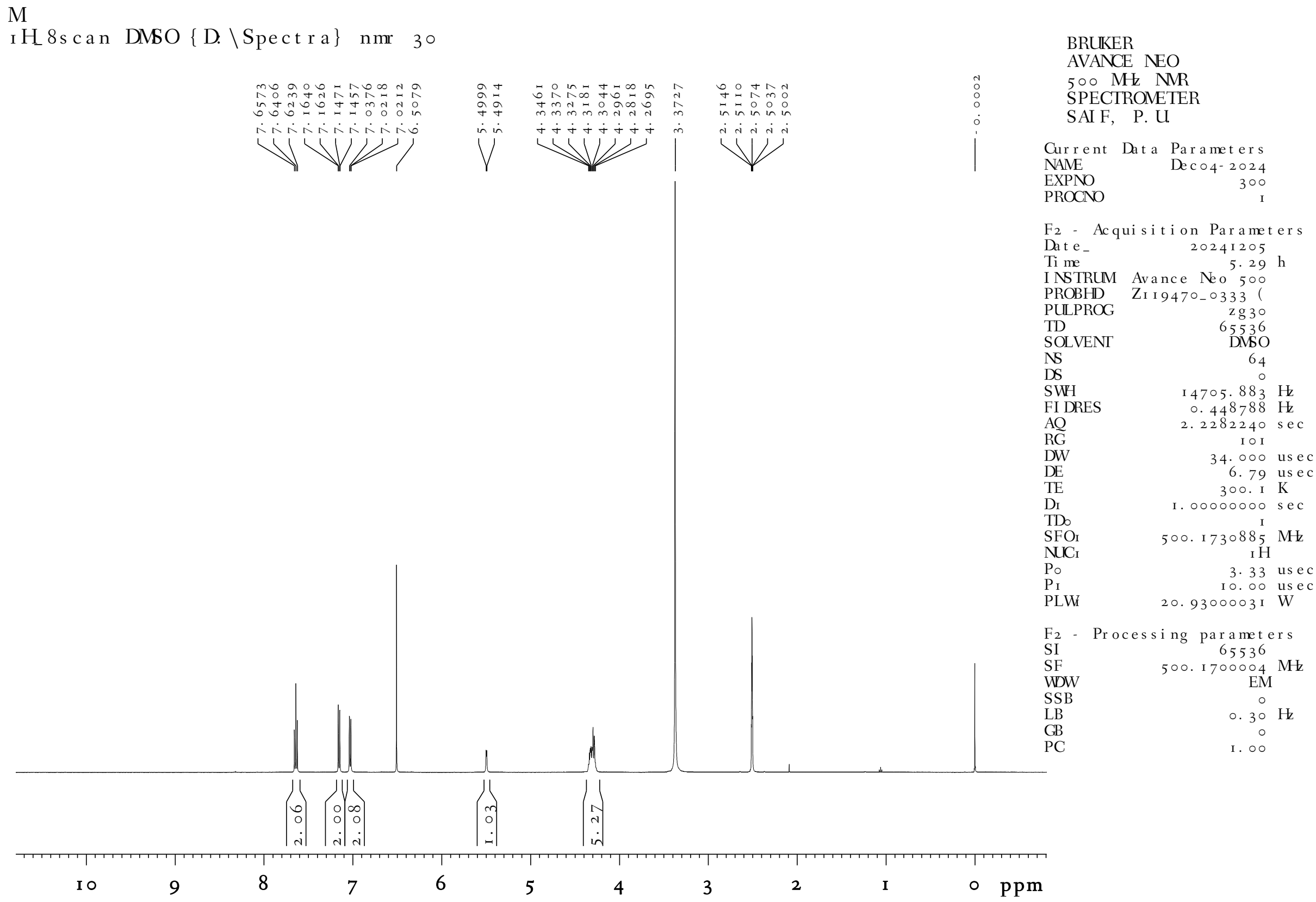

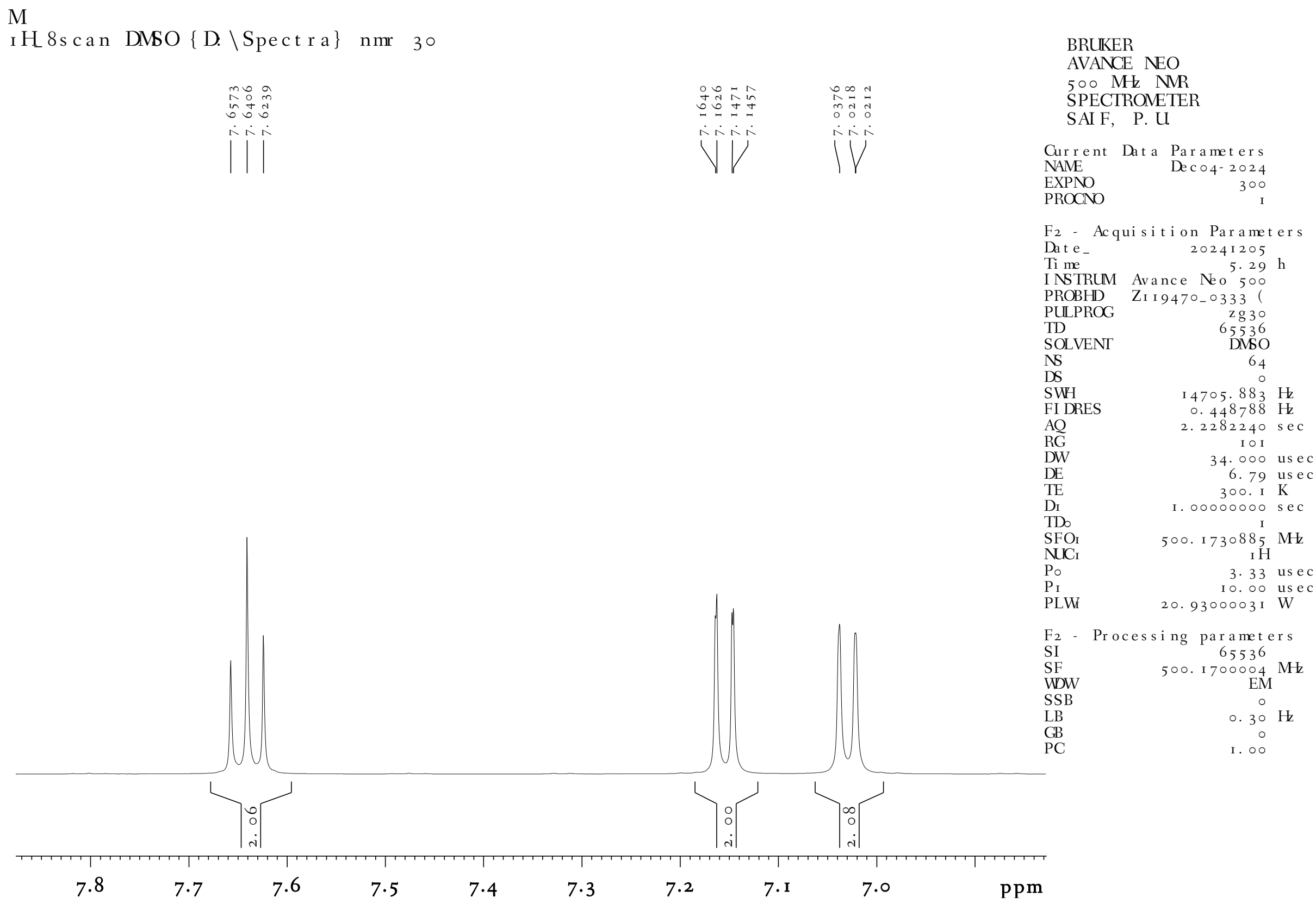

M
1H_8scan DMSO {D:\Spectra} nmr 30

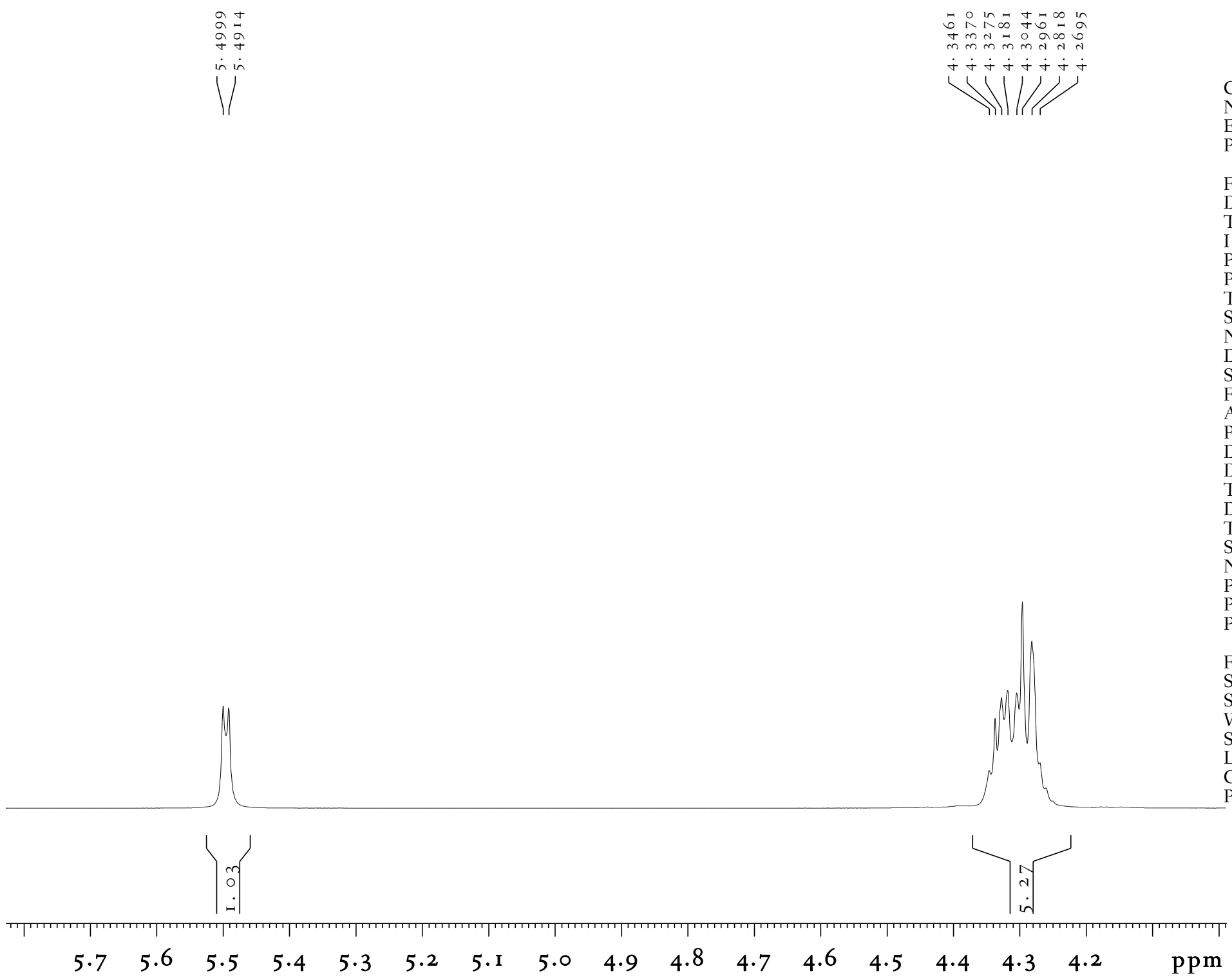

BRUKER
AVANCE NEO
500 MHz NMR
SPECTROMETER
SAIF, P.U

Current Data Parameters

NAME	Dec04-2024
EXPNO	300
PROCNO	1

F2 - Acquisition Parameters

Date_	20241205
Time	5.29 h
INSTRUM	Avance Neo 500
PROBHD	Z119470_0333 (
PULPROG	zg30
TD	65536
SOLVENT	DMSO
NS	64
DS	0
SWH	14705.883 Hz
FIDRES	0.448788 Hz
AQ	2.2282240 sec
RG	101
DW	34.000 usec
DE	6.79 usec
TE	300.1 K
D1	1.00000000 sec
TD0	1
SFO1	500.1730885 MHz
NUC1	1H
P0	3.33 usec
P1	10.00 usec
PLW1	20.93000031 W

F2 - Processing parameters

SI	65536
SF	500.1700004 MHz
WDW	EM
SSB	0
LB	0.30 Hz
GB	0
PC	1.00
