# Supplementary material for: Dual antimicrobial and anti-inflammatory efficacy of cetylpyridinium–cromolyn ion-pair nanoparticles delivered via modified gelatin
Source: RSC Adv. 2026 Nov 4. Online ahead of print. doi: 10.1039/d6ra07879f (PMC13732725; doi:10.1039/d6ra07879f)
Supplement: Supplementary file 2 [file RA-OLF-D6RA07879F-s003.pdf]

Supplementary Information (SI) for RSC Advances.
This journal is © The Royal Society of Chemistry

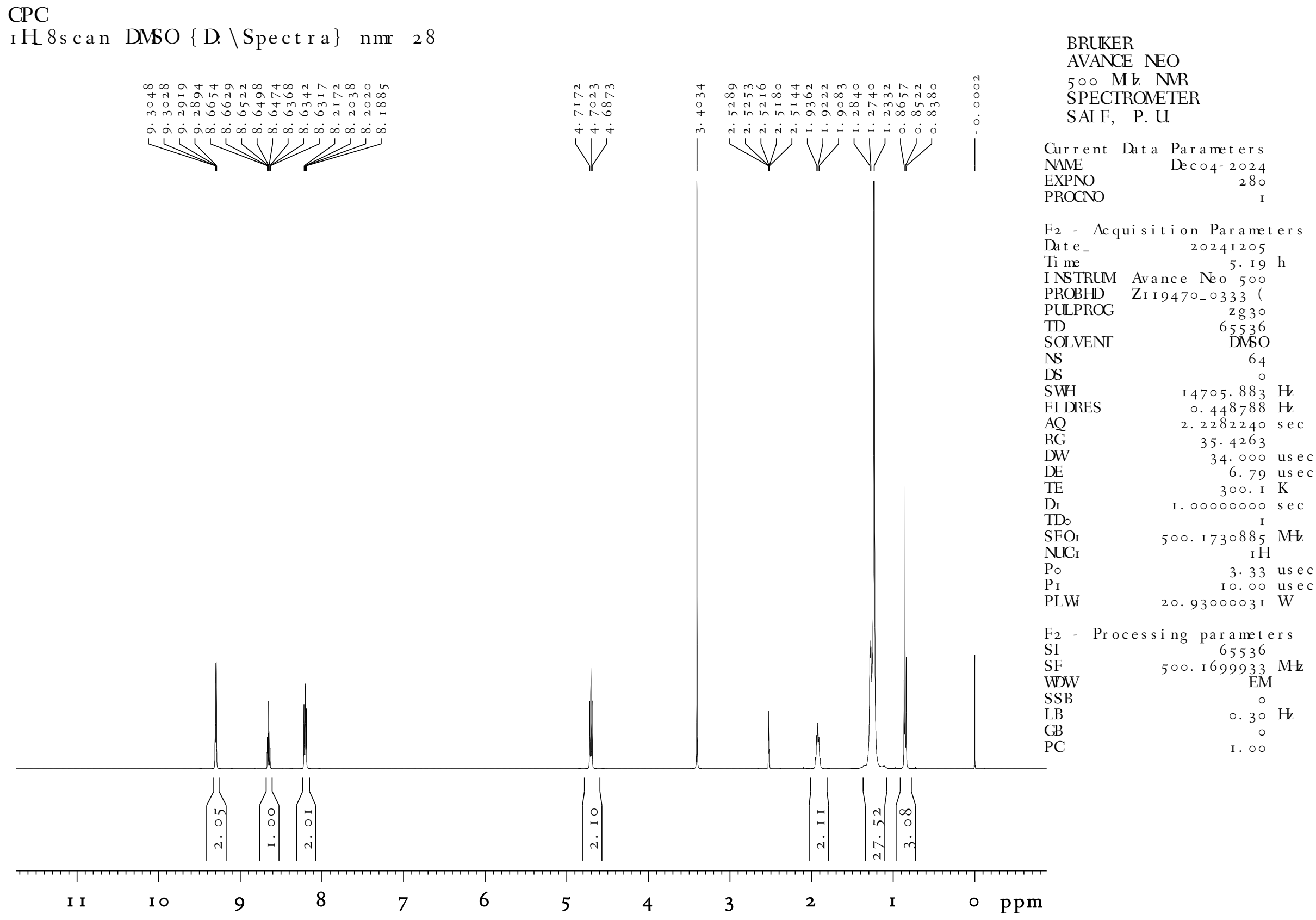

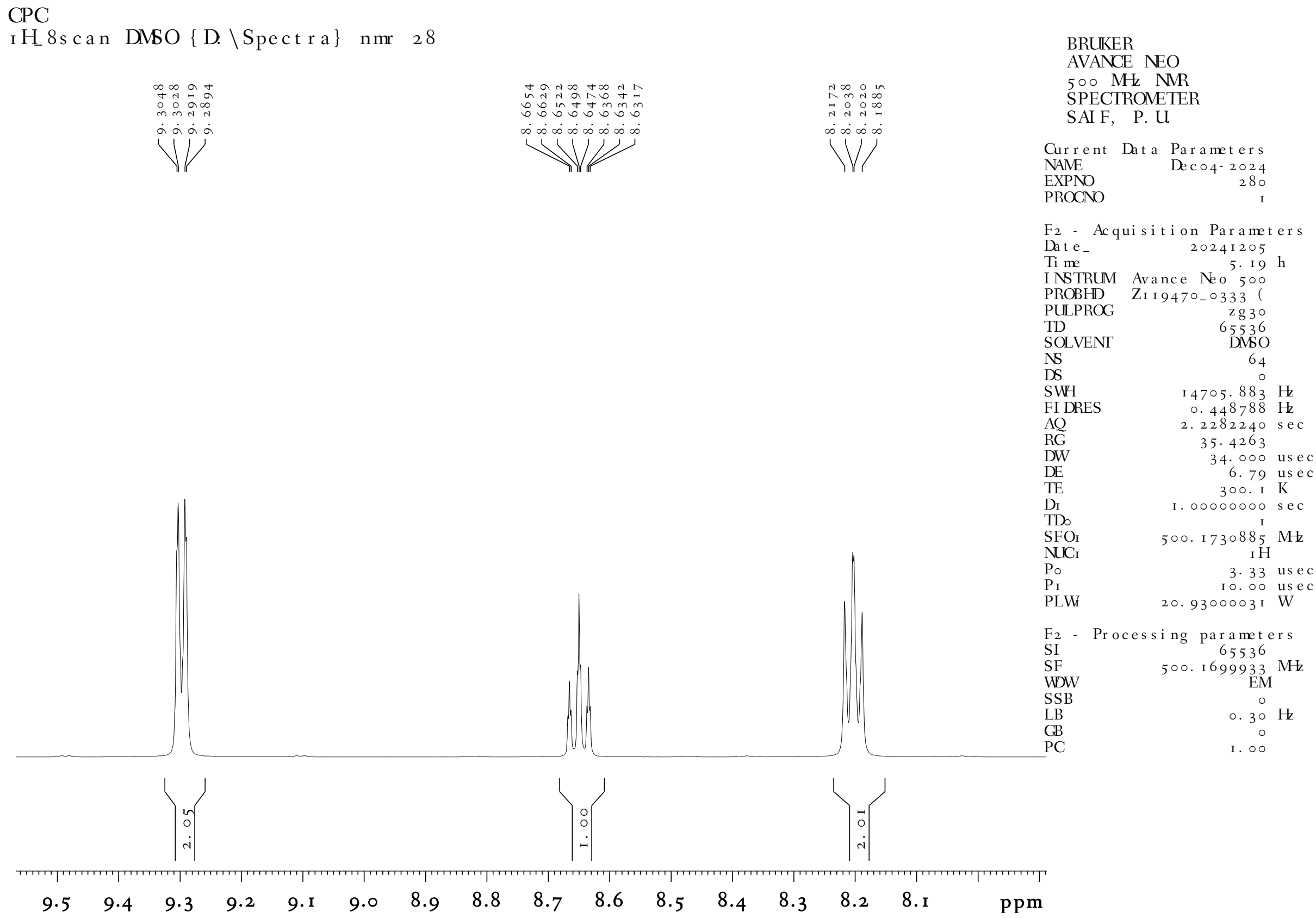

CPC
1H_8scan DMSO {D:\Spectra} nmr 28

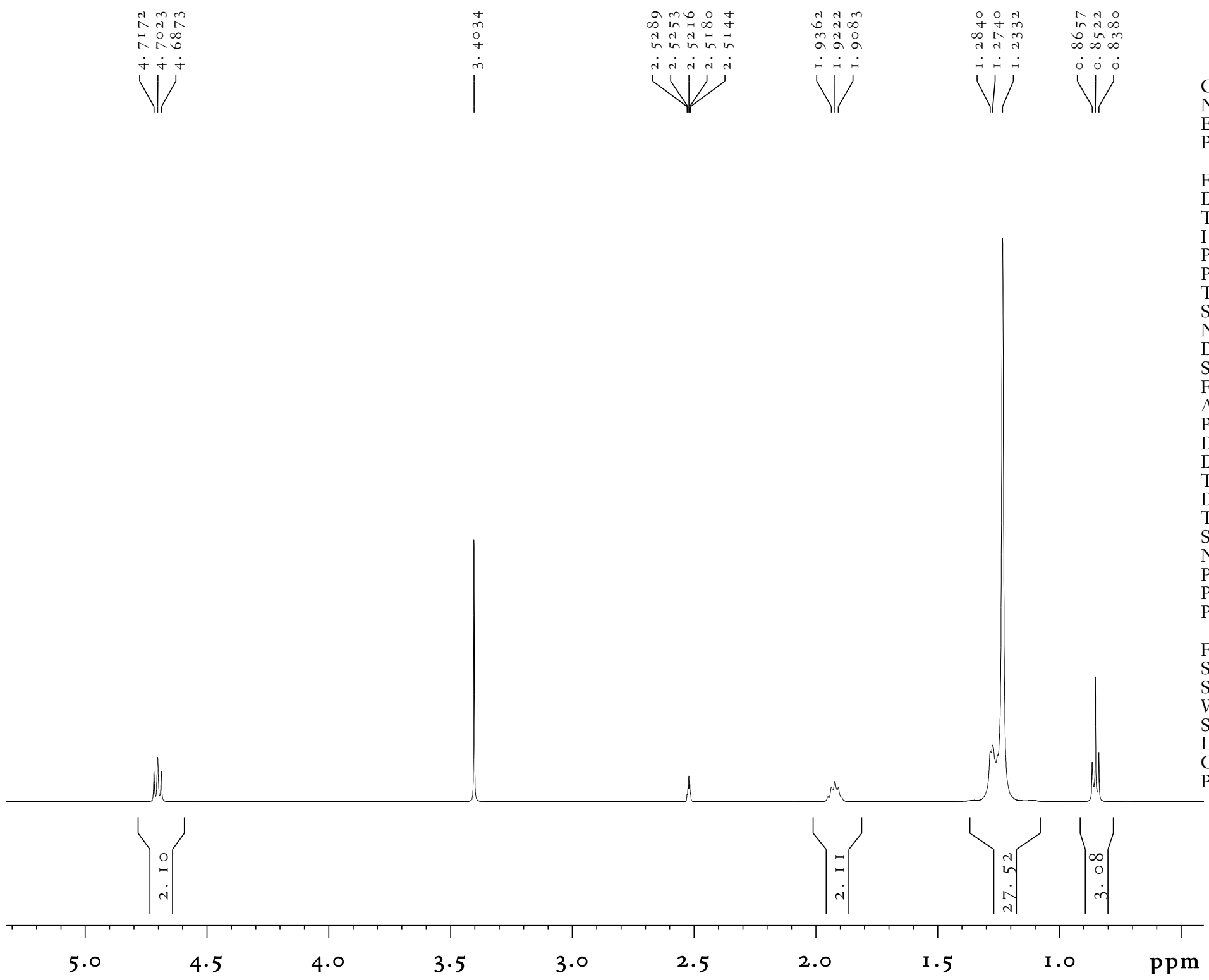

BRUKER
AVANCE NEO
500 MHz NMR
SPECTROMETER
SAIF, P.U

Current Data Parameters
NAME Dec04-2024
EXPNO 280
PROCNO 1

F2 - Acquisition Parameters
Date_ 20241205
Time 5.19 h
INSTRUM Avance Neo 500
PROBHD Z119470_0333 (
PULPROG zg30
TD 65536
SOLVENT DMSO
NS 64
DS 0
SWH 14705.883 Hz
FIDRES 0.448788 Hz
AQ 2.2282240 sec
RG 35.4263
DW 34.000 usec
DE 6.79 usec
TE 300.1 K
D1 1.00000000 sec
TD0 1
SFO1 500.1730885 MHz
NUC1 1H
P0 3.33 usec
P1 10.00 usec
PLW1 20.93000031 W

F2 - Processing parameters
SI 65536
SF 500.1699933 MHz
WDW EM
SSB 0
LB 0.30 Hz
GB 0
PC 1.00
