# Supplementary material for: Dual antimicrobial and anti-inflammatory efficacy of cetylpyridinium–cromolyn ion-pair nanoparticles delivered via modified gelatin
Source: RSC Adv. 2026 Nov 4. Online ahead of print. doi: 10.1039/d6ra07879f (PMC13732725; doi:10.1039/d6ra07879f)
Supplement: Supplementary file 3 [file RA-OLF-D6RA07879F-s004.pdf]

Supplementary Information (SI) for RSC Advances.
This journal is © The Royal Society of Chemistry

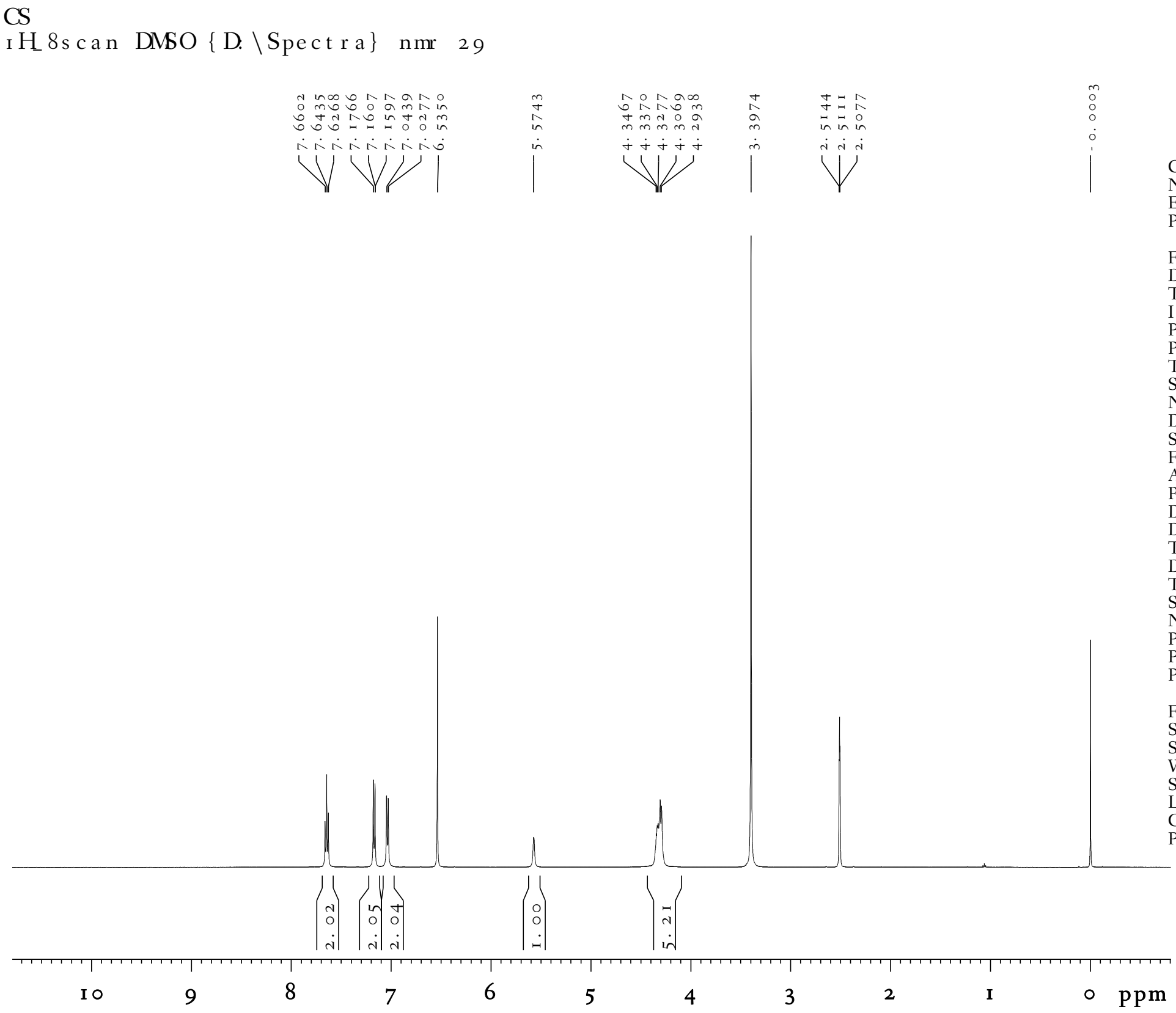

BRUKER
AVANCE NEO
500 MHz NMR
SPECTROMETER
SAIF, P.U

Current Data Parameters
NAME Dec04-2024
EXPNO 290
PROCNO 1

F2 - Acquisition Parameters
Date_ 20241205
Time 5.24 h
INSTRUM Avance Neo 500
PROBHD Z119470_0333 (
PULPROG zg30
TD 65536
SOLVENT DMSO
NS 64
DS 0
SWH 14705.883 Hz
FIDRES 0.448788 Hz
AQ 2.2282240 sec
RG 95.7854
DW 34.000 usec
DE 6.79 usec
TE 300.2 K
D1 1.00000000 sec
TD0 1
SFO1 500.1730885 MHz
NUC1 1H
P0 3.33 usec
P1 10.00 usec
PLW1 20.93000031 W

F2 - Processing parameters
SI 65536
SF 500.1699984 MHz
WDW EM
SSB 0
LB 0.30 Hz
GB 0
PC 1.00

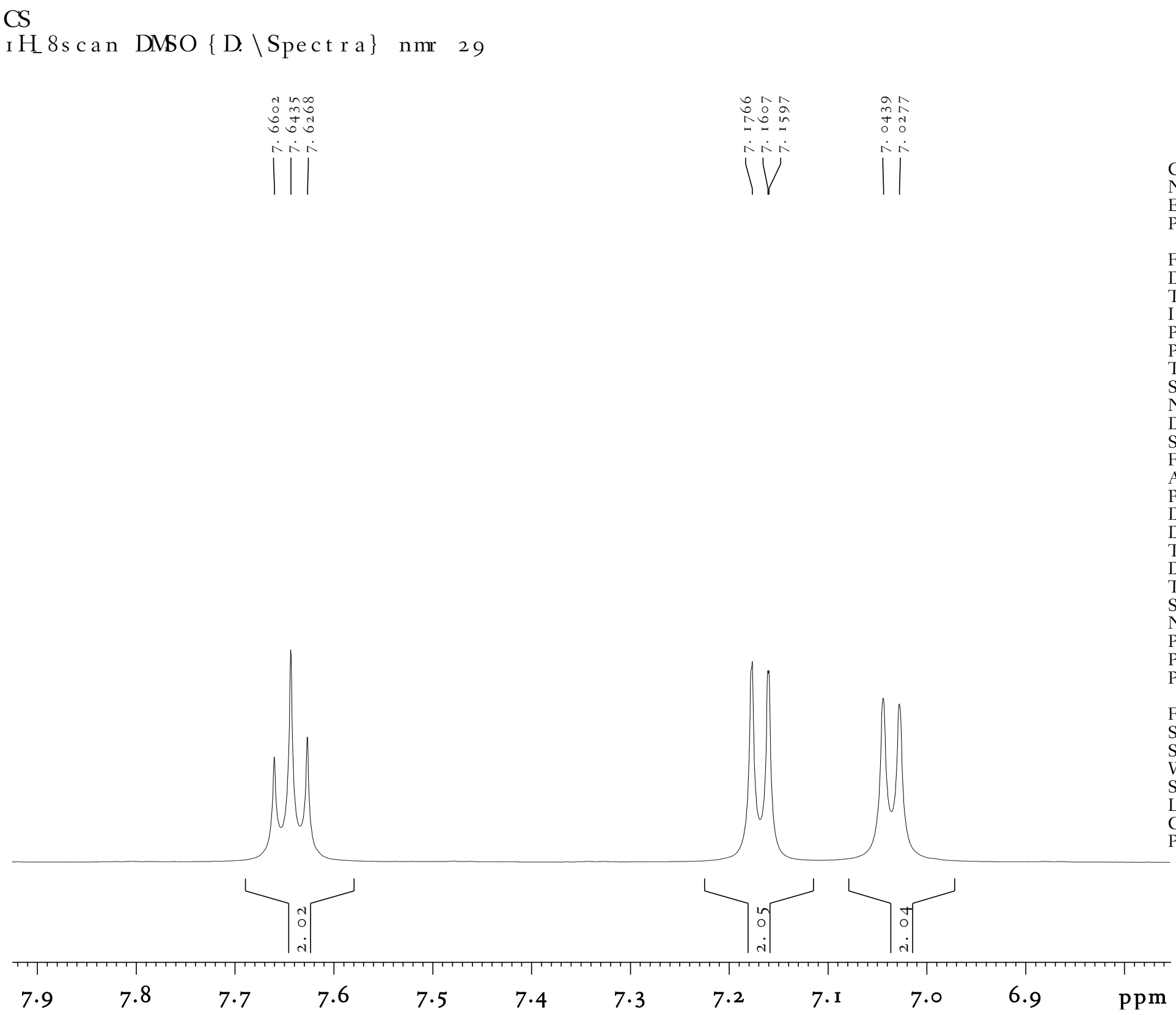

BRUKER
AVANCE NEO
500 MHz NMR
SPECTROMETER
SAIF, P.U

Current Data Parameters
NAME Dec04-2024
EXPNO 290
PROCNO 1

F2 - Acquisition Parameters
Date_ 20241205
Time 5.24 h
INSTRUM Avance Neo 500
PROBHD Z119470_0333 (
PULPROG zg30
TD 65536
SOLVENT DMSO
NS 64
DS 0
SWH 14705.883 Hz
FIDRES 0.448788 Hz
AQ 2.2282240 sec
RG 95.7854
DW 34.000 usec
DE 6.79 usec
TE 300.2 K
D1 1.00000000 sec
TD0 1
SFO1 500.1730885 MHz
NUC1 1H
P0 3.33 usec
P1 10.00 usec
PLW1 20.93000031 W

F2 - Processing parameters
SI 65536
SF 500.1699984 MHz
WDW EM
SSB 0
LB 0.30 Hz
GB 0
PC 1.00

CS
1H_8scan DMSO {D:\Spectra} nmr 29

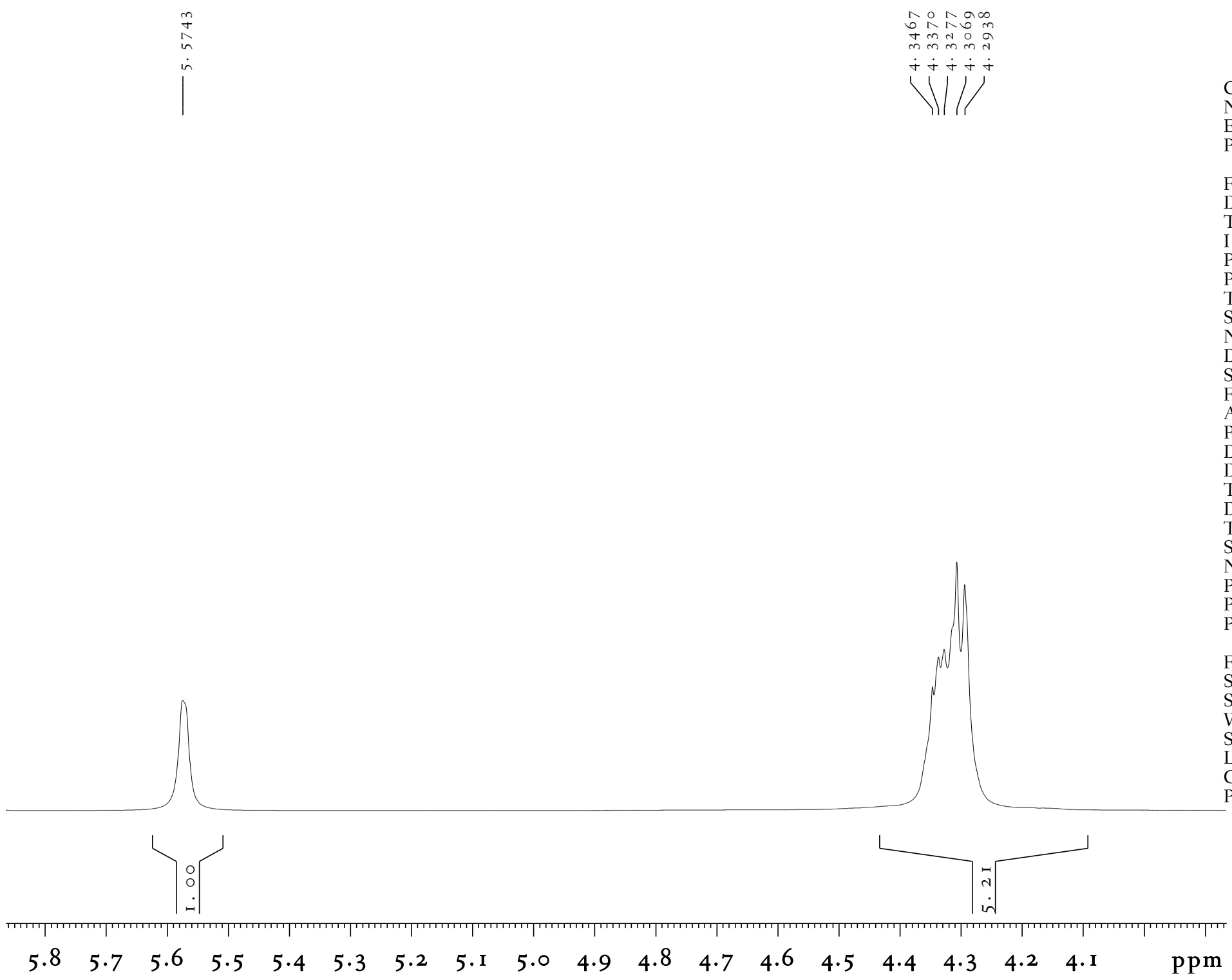

BRUKER
AVANCE NEO
500 MHz NMR
SPECTROMETER
SAIF, P.U

Current Data Parameters

NAME	Dec04-2024	
EXPNO	290	
PROCNO	1	

F2 - Acquisition Parameters

Date_	20241205	
Time	5.24	h
INSTRUM	Avance Neo 500	
PROBHD	Z119470_0333 (	
PULPROG	zg30	
TD	65536	
SOLVENT	DMSO	
NS	64	
DS	0	
SWH	14705.883	Hz
FIDRES	0.448788	Hz
AQ	2.2282240	sec
RG	95.7854	
DW	34.000	usec
DE	6.79	usec
TE	300.2	K
D1	1.00000000	sec
TD0	1	
SFO1	500.1730885	MHz
NUC1	1H	
P0	3.33	usec
P1	10.00	usec
PLW1	20.93000031	W

F2 - Processing parameters

SI	65536	
SF	500.1699984	MHz
WDW	EM	
SSB	0	
LB	0.30	Hz
GB	0	
PC	1.00	
